# Increased Long Chain acyl-Coa Synthetase Activity and Fatty Acid Import Is Linked to Membrane Synthesis for Development of Picornavirus Replication Organelles

**DOI:** 10.1371/journal.ppat.1003401

**Published:** 2013-06-06

**Authors:** Jules A. Nchoutmboube, Ekaterina G. Viktorova, Alison J. Scott, Lauren A. Ford, Zhengtong Pei, Paul A. Watkins, Robert K. Ernst, George A. Belov

**Affiliations:** 1 Virginia-Maryland Regional College of Veterinary Medicine, University of Maryland, College Park, Maryland, United States of America; 2 University of Maryland, School of Dentistry, Baltimore, Maryland, United States of America; 3 Kennedy Krieger Institute and Johns Hopkins University School of Medicine, Baltimore, Maryland, United States of America; University of California at Irvine, United States of America

## Abstract

All positive strand (+RNA) viruses of eukaryotes replicate their genomes in association with membranes. The mechanisms of membrane remodeling in infected cells represent attractive targets for designing future therapeutics, but our understanding of this process is very limited. Elements of autophagy and/or the secretory pathway were proposed to be hijacked for building of picornavirus replication organelles. However, even closely related viruses differ significantly in their requirements for components of these pathways. We demonstrate here that infection with diverse picornaviruses rapidly activates import of long chain fatty acids. While in non-infected cells the imported fatty acids are channeled to lipid droplets, in infected cells the synthesis of neutral lipids is shut down and the fatty acids are utilized in highly up-regulated phosphatidylcholine synthesis. Thus the replication organelles are likely built from *de novo* synthesized membrane material, rather than from the remodeled pre-existing membranes. We show that activation of fatty acid import is linked to the up-regulation of cellular long chain acyl-CoA synthetase activity and identify the long chain acyl-CoA syntheatse3 (Acsl3) as a novel host factor required for polio replication. Poliovirus protein 2A is required to trigger the activation of import of fatty acids independent of its protease activity. Shift in fatty acid import preferences by infected cells results in synthesis of phosphatidylcholines different from those in uninfected cells, arguing that the viral replication organelles possess unique properties compared to the pre-existing membranes. Our data show how poliovirus can change the overall cellular membrane homeostasis by targeting one critical process. They explain earlier observations of increased phospholipid synthesis in infected cells and suggest a simple model of the structural development of the membranous scaffold of replication complexes of picorna-like viruses, that may be relevant for other (+)RNA viruses as well.

## Author Summary

Eukaryotic cells feature astonishing complexity of regulatory networks, yet control over this fine-tuned machinery is easily overrun by viruses with expression of just a handful of proteins. One of the striking examples of such hostile take-over is the rewiring of normal cellular membrane metabolism by (+)RNA viruses towards development of new membranous organelles harboring viral replication machinery. (+)RNA viruses of eukaryotes infect organisms from unicellular algae to humans. Many of them induce diseases resulting in significant economic losses, public health burden, human suffering and sometimes fatal consequences. We show how picornaviruses reorganize cellular lipid metabolism by targeting long chain acyl-CoA synthetase activity. This induces increased import of fatty acids in infected cells and up-regulation of phospholipid synthesis, resulting in formation of replication organelles different from the pre-existing cellular membranes. This mechanism is utilized by diverse viruses and may represent an attractive target for anti-viral interventions.

## Introduction

(+)RNA viruses of eukaryotes are a very successful group of pathogens infecting organisms from unicellular algae to humans. In spite of adaptation to diverse hosts the basic processes of genome expression and replication are highly conserved among these viruses. One such feature shared among all (+)RNA viruses is the association of RNA replication machinery with cellular membranes. It has been proposed that assembly of replication complexes on membranes may facilitate infection in several ways: increase local concentration of viral proteins; provide structural scaffold for assembly of replication machinery; hide viral dsRNA replication intermediates from the cellular innate immunity mechanisms (reviewed in [1], [2]).

Poliovirus (PV) is a prototype species of the *Picornaviridae* family. Its genome RNA of about 7500 nucleotides is directly translated into one polyprotein which is cleaved co- and post-translationally into a dozen of structural and replication proteins. Proteins encoded in the P2-P3 region of the viral genome as well as the intermediate products of the polyprotein processing are responsible for RNA replication. Other members of the *Picornaviridae* family share the same basic genome organization and expression strategy with minor modifications [3].

PV infection induces rapid development of new membranous agglomerates harboring viral replication complexes. The current models of the development of picornavirus replication structures suggest hijacking of either elements of the cellular secretory pathway or autophagy machinery [4], [5], [6]. However even closely related viruses vary greatly in their sensitivity to the inhibitors of the secretory pathway, and effects of manipulation of autophagy may vary even for the same virus [7], [8], [9], suggesting that these cellular processes are not obligatory for the development of replication complexes. At the same time previously accumulated data show that diverse picornaviruses similarly induce strong stimulation of phospholipid biosynthesis, especially phosphatidylcholine (PC), upon infection with [10], [11], [12], [13].

PC constitutes about 50% of the total phospholipid content in eukaryotic membranes [14]. Phospholipids found in cellular membranes include fatty acids (FAs) with C16 and longer carbon atoms chains [15]. In mammalian cells fatty acid synthase can *de novo* synthetize palmitic acid (C16:0), which can subsequently be processed into other FA species [16], [17]. However, most of the cells import the majority of long chain FAs from extracellular media. The mechanism of FA transport through plasma membrane is not yet completely understood, however it is believed that acyl-CoA synthetase activity plays a key role in this process. According to the current model of vectorial acylation, long chain FAs as hydrophobic molecules can freely diffuse through lipid bilayers, and inside the cell they are converted into hydrophilic acyl-CoAs that can no longer escape. Indeed most proteins that have been shown to facilitate FA uptake possess acyl-CoA synthetase activity and its inactivation prevented transport of FAs into cells [18], [19], [20], [21]. Thus lipid biosynthesis is intrinsically dependent on acyl-CoA synthetases which activate FAs derived from either external or internal cellular sources.

There are 26 different acyl-CoA synthetase genes in human genome [22]. Five members of the long chain acyl-CoA synthetase (Acsl) family; six proteins of the very long chain acyl-CoA synthetase family also known as fatty acid transport proteins (Acsvl or FATP); and two bubblegum acyl-CoA synthetases (ACSBG) can activate long chain fatty acids. Their differential tissue expression and sub-cellular localization, existence of multiple splice isoforms, and enzymatic preference towards certain classes of FA provide foundation for complex pattern of uptake and channeling of FA into different metabolic pathways [23].

In this study we show that PV infection results in fast up-regulation of long chain FA uptake due to activation of cellular long chain acyl-CoA synthetase activity, and we identify long chain acyl-CoA synthetase 3 (Acsl3) as a novel host factor required for polio replication. We found that in mock-infected cells the newly-imported FAs are mostly channeled to lipid droplets, while in infected cells they are immediately utilized for highly up-regulated PC synthesis. The infected cells demonstrate preference for import of different FAs than mock-infected cells, resulting in significant changes in the spectrum of PC molecules. The enrichment of phosphatidylcholine species with short palmitoyl (C16:0) moieties likely generates more fluid membranes with intrinsic capacity to assemble into convoluted tubular matrix of the membranous replication organelles. We find that stimulation of FA import requires PV protein 2A, but is independent of its protease activity, thus revealing a new important function this protein plays in alteration of the cell metabolism. The activation of FA import is observed upon infection of diverse picornaviruses in different cell types. Our work explains previous data on stimulation of membrane synthesis and morphology of replication structures in picornavirus-infected cells, and provides a new model of the development of the membranous scaffold of the replication organelles apparently shared by diverse picornaviruses.

## Results

### Poliovirus infection increases cellular long chain FA import and prevents their targeting to lipid droplets

**Figure 1:**
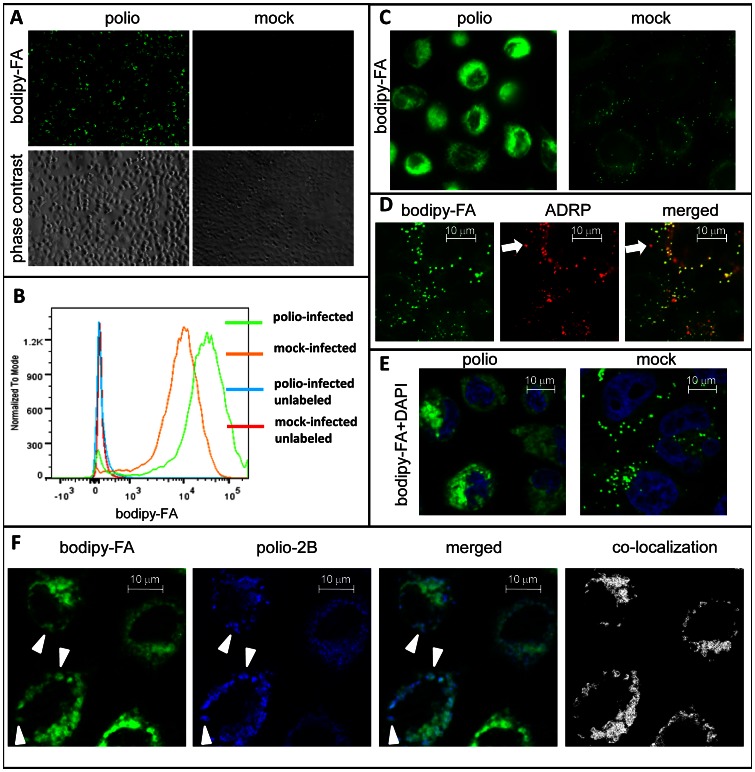
Poliovirus infection induces strong activation of fatty acid import. HeLa cells were infected with poliovirus at 50 PFU/cell, and at 4 h p. i. Bodipy 500/510 C4–C9 (bodipy-FA) was added for 30 min. **A**. low magnification view of infected vs mock-infected cells. **B**. FACS analysis of the fluorescence of infected (mock-infected) cells after 30 min pulse label with bodipy-FA at 4 h p. I, or control samples incubated without bodipy-FA. **C**. Higher magnification image showing that bodipy-FA probe is redistributed preferentially into lipid droplets in mock-infected cells and into membranes in infected cells. **D**. A confocal image of HeLa cells expressing pmCherry-ADRP protein (a marker for lipid droplets) and labeled with bodipy-FA for 30 min. Arrow marks a lipid droplet that did not accumulate the newly-synthesized lipids during the labeling period and also indicate lack of bodipy fluorescence leakage into the red channel. **E**. A confocal image of infected (mock-infected) HeLa cells labeled at 4 h p. i. with bodipy-FA and Hoechst-33342 (cell permeable DNA stain) for 30 min showing localization of bodipy-FA staining. **F**. A confocal image of polio-infected HeLa cells incubated for 30 min with bodipy-FA at 4 h.p.i. and processed for staining for a viral membrane-targeted protein 2B showing co-localization of the viral antigen and imported FA. Colocalization panel shows colocalized green and blue pixels identified with ImageJ software.

The increase of phospholipid synthesis in PV-infected cells [10], [12], [13] should be sustained by sufficient supply of corresponding precursors including long chain FAs. To monitor FA import we pulse-labeled PV-infected HeLa cells with a fluorescent fatty acid Bodipy 500/510 C4, 9 (bodipy-FA) which is believed to mimic FA with 18 carbon atoms backbone. This and similar molecules are extensively used in lipid metabolism research and it was previously shown to be rapidly utilized by cellular lipid synthesis machinery and incorporated into phospholipids, triglycerides and other natural lipids [24], [25], [26], [27], [28]. The cells were infected at a multiplicity of 50 PFU/cell to ensure simultaneous development of infection, and bodipy-FA was added for 30 min at 4 hours post infection (h p.i.), in the middle of the infectious cycle. The infected cells showed strongly increased import of bodipy-FA (Fig. 1A and B). In mock-infected cells the label was distributed into multiple round structures in the cytoplasm and was also found in intracellular ER-like staining (Fig. 1C, mock). The round bright dots were identified as lipid droplets since they co-localized with a well-established lipid droplet marker ADRP [29] (Fig. 1D). Note that some ADRP-positive structures did not accumulate bodipy-FA during 30 min labeling period (Fig. 1D, arrow), consistently with the previous results that individual lipid droplets accumulate newly-synthesized lipids at different rates [30]. In infected cells, bright bodipy-FA fluorescence surrounded the nuclei and often occupied the total cytoplasmic area reflecting robust development of the poliovirus replication complexes (note pycnotic nuclei in infected cells, characteristic of polio-induced cytopathic effect)(Fig. 1E). For the experiment shown on Fig. 1 the cells were incubated in serum-free media during the labeling period, so bodipy-FA was the only fatty acid available exogenously. We also monitored FA transport when cells were incubated in normal growth media supplemented with fetal bovine serum which provides ample supply of natural FA and other lipids. As expected, the level of fluorescent signal was lower in the presence of serum, due to competition with the fatty acids from serum, but the overall picture of strong stimulation of fatty acid import upon infection was the same (not shown). Cells on Figure 1C and E are imaged directly after formaldehyde fixation without further detergent permeabilization which we found to deteriorate the fine structure of the distribution of the bodipy-FA label, especially in weakly labeled mock-infected cells. Staining of cells for a viral antigen 2B, a marker of membranous replication complexes, revealed extensive co-localization of bodipy-FA fluorescence with polio replication structures, especially in the cells where viral protein staining could still be visualized as discrete domains in the confocal plain (Fig. 1F, arrowheads, also co-localization panel). With the further development of infection staining for both viral proteins and bodipy-FA tend to occupied all available perinuclear space reflecting massive development of membranous replication complexes. Staining for other membrane-targeted poliovirus replication proteins 2C and 3A revealed similar pattern of distribution of a viral antigen and bodipy-FA label (not shown).

Thus in PV-infected cells the import of FAs from media is strongly increased, their intracellular targeting is different from mock-infected cells, and they are used for building of viral membranous replication complexes.

### Imported FAs are directed to highly increased phosphatidylcholine synthesis in infected cells

**Figure 2:**
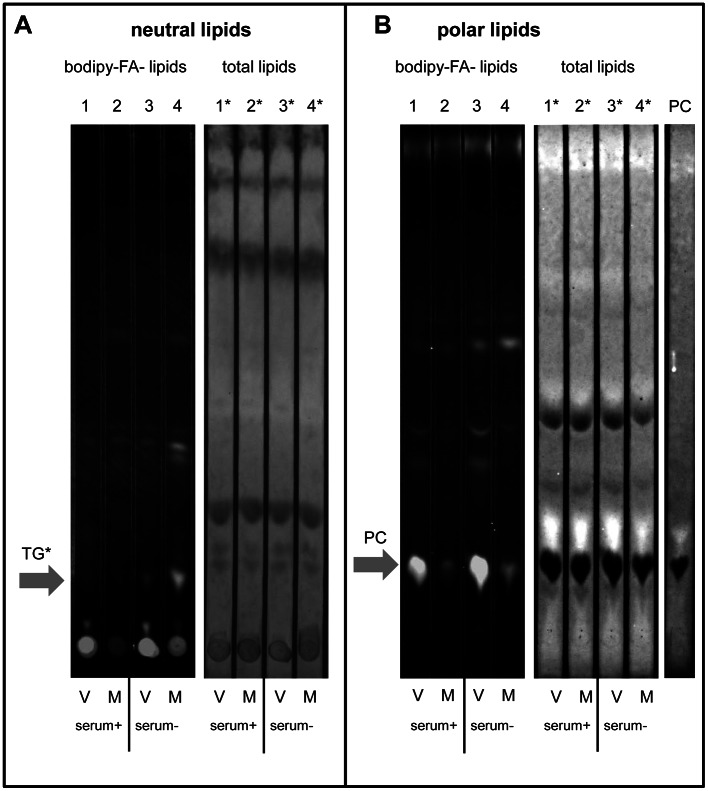
In infected cells synthesis of neutral lipids is shut down and imported fatty acids are directed to strongly stimulated synthesis of phosphatidylcholine. HeLa cells were infected (V) or mock-infected (M) with poliovirus at 50 PFU/cell and incubated in medium with or without serum as indicated. Bodipy-FA was added for 30 min at 4 h p. i.; after that total lipids were extracted and resolved by thin layer chromatography. **A**. Neutral lipids resolved in hexane∶ether∶acetic acid (80∶20∶1) system. Bodipy-FA-lipids represent fluorescent fatty acid-containing lipids synthesized during 30 min labeling period. Total lipids show all neutral lipids stained with bromothymol. **B**. Polar lipids resolved in chloroform∶ethanol∶water∶triethylamine (30∶35∶7∶35) system. Bodipy-FA-lipids represent fluorescent fatty acid-containing lipids synthesized during 30 min labeling period. Total lipids show all phospholipids stained with Phostain. PC: 2-Oleoyl-1-palmitoyl-sn-glycero-3-phosphocholine (phosphatidylcholine marker).

To investigate the metabolic targeting of the imported FAs we pulse-labeled cells with bodipy-FA for 30 min at 4 h p. i., extracted the lipids and resolved them by thin layer chromatography (TLC) using solvent systems optimized for separation of either neutral or polar lipids. The chromatograms were first photographed in a fluorescence imager to reveal the newly synthesized lipids, and then developed with conventional stains to visualize the total lipid material. We did not recover noticeable amount of free bodipy-FA. Virtually all the fluorescence was found in newly synthesized complex lipids, thus validating the use of bodipy-FA in our system (Figure S1, compare bodipy-FA marker lane 8 on the fluorescent polar lipids panel with the fluorescent lipids extracted from the cells (lanes 1–4 on the same panel)). There were no dramatic differences due incubation of cells in the presence or absence of serum, but as expected the fluorescent signal recovered from the serum-free samples was higher, about 1.5× for mock-infected cells and ∼2× for virus-infected cells as quantitated from the fluorescence of the total lipid spots loaded at the start position before TLC resolution (not shown). In the mock-infected cells incubated without serum significant amount of fluorescent label appeared in a spot on neutral lipids plate likely representing triglycerides with abnormal mobility due to the presence of bodipy-FA (Fig. 2A, lane 4), correlating with the microscopy observation of fluorescence accumulation in lipid droplets. Note that free bodipy-FA in the neutral lipid separation system moved much slower than the long chain free FA markers, while in the polar lipid separation system its mobility was close to C18 long chain free FAs (Figure S1, compare lanes 8 (free bodipy-FA, white horizontal arrows) and 5 (stearic acid C18:0), 6 (palmitic acid C16:0), 7 (linoleic acid C18:2) on neutral and polar lipid plates). We cannot exclude that this spot represents some other type of neutral lipid, but in any case synthesis of this compound is active in mock-infected cells and shut down in infected cells. In the lipids isolated from infected cells almost no fluorescence was resolved in the non-polar system, (Fig. 2A compare lanes 3 and 4), indicating that synthesis of neutral lipids is shut down. In fact in the TLC system optimized for neutral lipids most of the labeled lipids isolated from infected cells remained as a bright spot at the loading position (Fig. 2A, lanes 1 and 3). In contrast, on the TLC plate resolved using the polar solvent system we observed a very strong signal for newly synthesized PC in infected cells (Fig. 2B, compare lanes 1, 3 and 2, 4). The staining of the TLC plates for total lipid content did not reveal significant differences between infected and mock-infected cells (Fig. 2). It should be noted that the samples were analyzed after 4 h p. i., meaning that the period of up-regulated synthesis of new lipids was relatively short, and they apparently did not significantly change the overall lipid content of the cells. Thus, PV infection does not only increase the level of FA import but modifies their metabolic channeling by down-regulating synthesis of neutral lipids, and redirecting the newly imported FAs for the highly up-regulated production of PC.

### Long chain acyl-CoA synthetase activity in infected cells is up-regulated and exhibits substrate preference towards shorter FA

**Figure 3:**
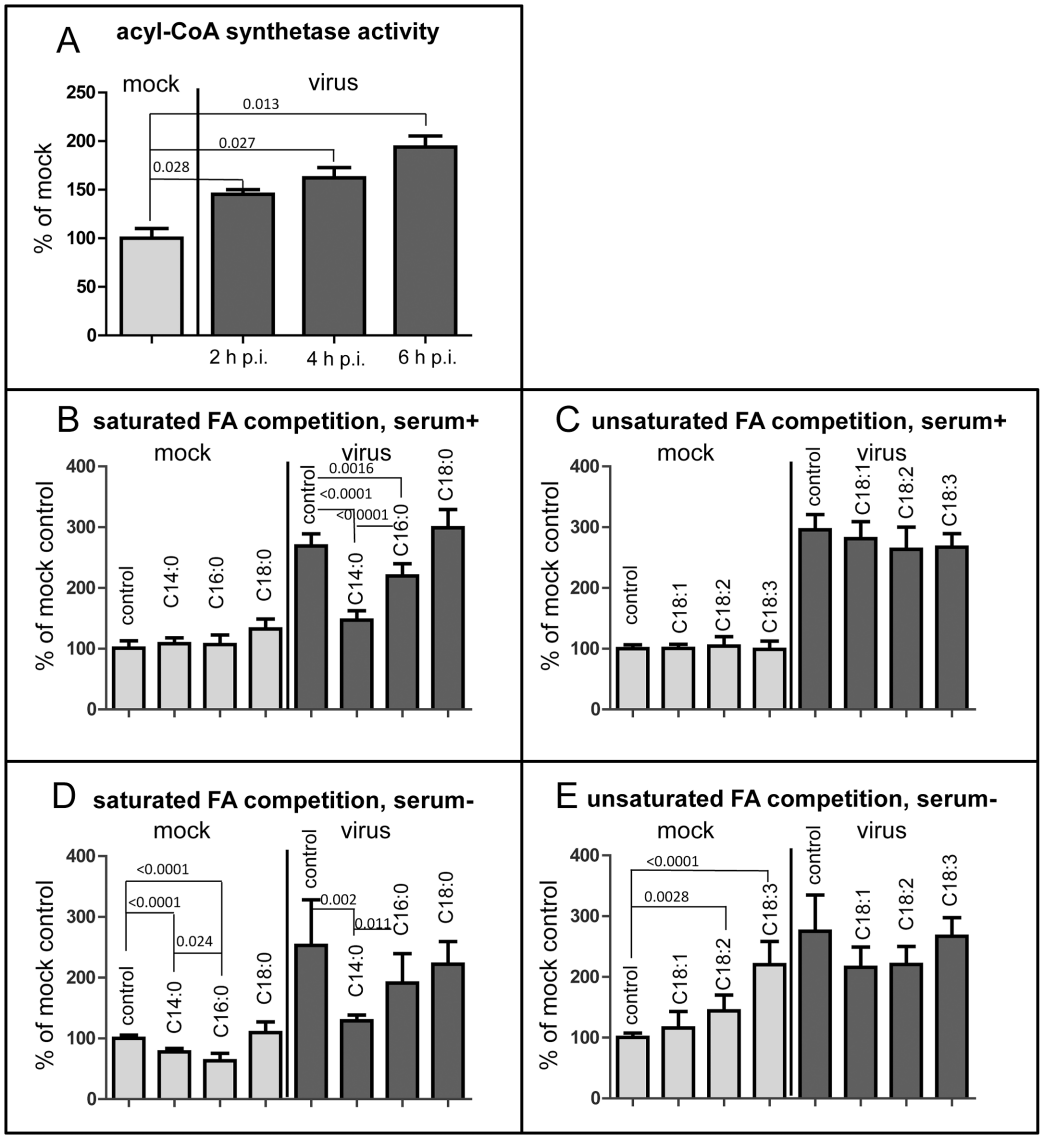
Modulation of long chain acyl-CoA synthetase activity in poliovirus-infected cells. **A**. Acyl-CoA synthetase activity is stimulated as early as 2 h p. i. and continues to increase during the time-course of infection. Acyl-CoA synthetase activity *in vitro* assay was performed with lysates of HeLa cells collected at indicated times post infection, the data are normalized to the activity of the lysate from mock-infected collected at 2 h p. i., p-values are shown. **B. and C**. Fatty import competition assay performed with the cells incubated in serum- supplemented medium during the whole experiment. HeLa cells were infected (or mock-infected) with poliovirus at 50 PFU/cell; at 4 h.p.i. bodipy-FA was added for 30 min in the presence of 125× molar excess of the indicated long chain fatty acids. No competitor fatty acid was added to control samples. The data are normalized to the signal from the mock-infected control sample; p-values are shown. **D. and E**. Fatty import competition assay performed with the cells incubated in serum-free medium during the whole experiment. HeLa cells were infected (or mock-infected) with poliovirus at 50 PFU/cell; at 4 h.p.i. bodipy-FA was added for 30 min in the presence of 125× molar excess of the indicated long chain fatty acids. No competitor fatty acid was added to control samples. The data are normalized to the signal from the mock-infected control sample; p-values indicating significant differences are shown.

Import of FAs is inextricably connected to activity of acyl-CoA synthetases [23]. Transport of saturated or unsaturated long-chain fatty acids containing 18 or fewer carbons across biological membranes is rapid and not thought to be rate-limiting [31], [32]. Thus the increased uptake of FFA probe suggests that long chain acyl-CoA synthetase activity must be up-regulated upon infection. To measure this activity we prepared lysates from HeLa cells infected at the multiplicity of 50 PFU/cell and incubated without serum for different times post-infection. The infected cells demonstrated elevated level of acyl-CoA synthetase activity as early as 2 h p.i which steadily increased at later times (Fig. 3A).

Cellular acyl-CoA synthetases have different preferences for the backbone length and degree of saturation of FA, although the substrate specificity is generally not very strict and one enzyme can activate multiple FA species [23], [33]. To assess the substrate specificity of acyl-CoA synthetases activated upon infection we performed FA import competition assay by labeling the cells at 4 h p. i. with bodipy-FA in the presence of 125× molar excess of competitor FAs. If the competitor FA is a preferred substrate over the bodipy-FA probe, it should result in the corresponding reduction of fluorescence. The control samples showed that infected cells incorporated more than 250% of bodipy-FA relative to mock-infected cells, in agreement with microscopy and TLC data (Fig. 3 B–E). In mock-infected cells incubated with serum no FA tested showed significant influence on the incorporation of bodipy-FA, likely because of the substantial amount of FAs already present in serum (Fig. 3B, C). In mock-infected cells incubated without serum the strongest competition was shown by palmitic acid (C16:0) (∼37% of mock control) while myristic acid (C14:0) demonstrated a weaker effect (∼22% of mock control) (Fig. 3D). The addition of unsaturated FAs to mock-infected cells incubated without serum actually significantly enhanced incorporation of bodipy-FA, up to more than 100% in case of linolenic (C18:3) acid (Fig. 3E), indicating that they were stimulating FA uptake by the cells starved without exogenous FAs for 4 hours. The virus-infected cells showed a completely different pattern. Unsaturated FAs: oleic (C18:1), linoleic (C18:2) and linolenic (C18:3), mildly reduced bodipy-FA incorporation in the presence and in the absence of serum (Fig. 3C, E). Palmitic acid (C16:0) inhibited import of the fluorescent label in the presence of serum from ∼270% to ∼220%, and in the absence of serum from ∼250% to ∼190% (Fig. 3A and C).The strongest competitor for the bodipy-FA incorporation in infected cells was myristic acid (C14:0) inhibiting bodipy-FA incorporation in the presence of serum from ∼270% to ∼150% and from ∼250% to ∼130% in the absence of serum (Fig. 3B, D).

These data show that acyl-CoA synthetase activity in infected cells is strongly stimulated from the early time post infection and that its substrate preference is changed.

### Poliovirus-infected cells accumulate different molecular species of PC than non-infected cells

**Figure 4:**
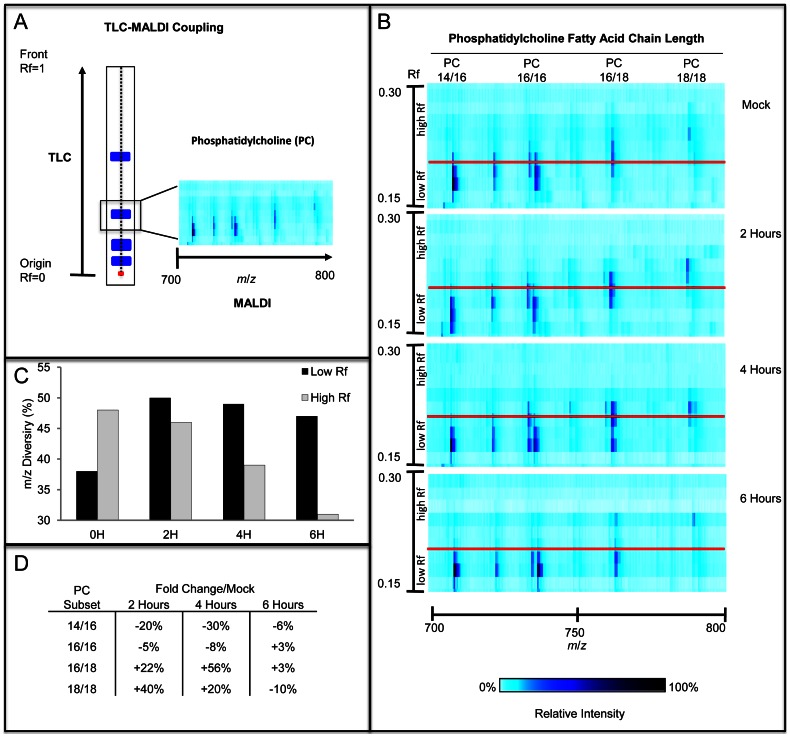
Shift in phosphatidylcholine spectrum following infection demonstrated by TLC-MALDI. **A**. Schematic representation of TLC-MALDI, blue spots represent phospholipid migration on TLC plate, hash marks represent stepwise MALDI data capture. **B**. HeLa cells were infected with poliovirus at 50 PFU/cell and processed for the total lipid extraction at the indicated time points post infection. TLC-MALDI data shown at phosphatidylcholine (PC) migration (Rf) range, fatty acid chain lengths noted, intensity of signal at respective mass to charge ratios (*m*/*z*) (blue scale). **C**. PC diversity (unique *m*/*z* signatures) shifts in abundance from higher Rf to lower Rf during time course of infection. **D**. Percent change of PC subset/PC total ratio compared to mock. Results from a representative experiment are shown.

The competition experiments suggest that the pool of acyl-CoAs available for new phospholipid synthesis should be different in infected and mock-infected cells. To investigate the changes in the spectrum of PC molecules, TLC-MALDI was used to couple the power of solvent resolution of phospholipids by TLC to the mass identification capacity of matrix assisted laser desorption-ionization time-of-flight mass spectrometry (MALDI-TOF-MS) (Fig. 4A). PC was first identified by characteristic TLC migration, and reflectron positive mode MALDI-TOF-MS was used to scan the TLC lane. The mass to charge ratio (*m*/*z*) was used to secondarily identify the major PC molecules and acyl variants (Fig. 4B). We observed a substantial drop in the diversity of the PC molecules containing long C18 chains, running in the high Rf chromatography zone, and correspondingly a rapid increase in the PCs with shorter acyl chains from the low Rf zone upon infection (Fig. 4C). The analysis of the individual PC classes demonstrates a fast shift in the composition of PCs during infection. At 2 h p. i. there is already a significant increase in PCs with C18/C18 acyl chains, as well as C16/C18 ones, accompanied by a noticeable drop in the abundance of the C14/C16 and C16/C16 PCs, compared to mock-infected cells. This general trend continues later in infection with an especially strong increase in the C16/C18 PC species at 4 h p. i. (Fig. 4D). Changes in lipid abundance at 6 h.p.i. do not follow the general trends observed at 2 and 4 h.p.i. likely due to the significant degree of cell lysis observed at this late stage of infection at high MOI. It should be noted that while the competition assay showed a strong preference for import of C14 myristic acid to infected cells, it only reflects the changes in the prevalent cellular acyl-CoA synthetase activity induced by polio infection, and cannot be directly interpreted as that myristic acid is the predominant imported FA in natural conditions. The actual composition of intracellular acyl-CoA pool will be shaped by the availability of the corresponding FA substrates. The resolution of TLC-MALDI is not sufficient to separate PC molecules with saturated and unsaturated FA chains with the same number of carbon atoms. Thus, PV infection does not only up-regulates the overall synthesis of PC but specifically changes the molecular composition of this structural phospholipid indicating that membranes of PV replication complexes are significantly different from the pre-existing cellular membranes.

### Increase of FA import in infected cells does not require new cellular genes expression and once activated is independent of viral RNA replication and translation

We investigated effect of different inhibitors of cellular metabolism and viral replication on the activation of FA import. The possibility that a PV protein(s) may have acyl-CoA synthetase activity is unlikely since all known acyl-CoA synthetases have signature of two conserved motifs [22] lacking in the PV polyprotein. PV replication proceeds in the cytoplasm and induces rapid shut-off of cellular mRNA translation, inhibition of nuclear transcription and disruption of nucleo-cytoplasmic barrier [3]. Indeed replication of poliovirus t. I Mahoney is not affected by actinomycin D (AMD), an inhibitor of nuclear transcription [34], [35], our observations (not shown). We assessed the effect of inhibition of cellular transcription on increase of fatty acids import upon polio infection. The cells were pre-incubated with AMD for 30 min before the infection, and the inhibitor was present in the media further on during the whole time of infection and bodipy-FA labeling. Consistent with the sufficiency of pre-existing cellular factors for poliovirus replication, we observed that actinomycin D had no effect on the activation of bodipy-FA import in infected cells (Figure S2, panels A and B). We also investigated if continuous synthesis of PV RNA and proteins are required to sustain the elevated FA uptake by infected cells. The infection was allowed to proceed normally for 3.5 h without the inhibitors, and then guanidine-HCl, a strong specific inhibitor of PV RNA replication [36], or cycloheximide, a general inhibitor of translation, were added. After 30 min incubation with the inhibitors, the medium was replaced with the labeling media that contained bodipy-FA and the corresponding inhibitors, and the cells were incubated for another 30 min. Thus the labeling was performed when synthesis of the viral macromolecules was already inhibited for 30 min. The experiment shows that inhibition of polio RNA and protein synthesis did not prevent enhanced import of fatty acids (Figure S2, C, D and E). Since infection in the control sample effectively proceeded an hour more than in the inhibitor-treated samples (30 min pre-incubation +30 min labeling in the presence of inhibitors), control infected cells show higher bodipy-FA accumulation, consistently with the correlation between the amount of viral proteins and the level of FA import stimulation.

Thus, increase of FA import in infected cells does not depend on new expression of cellular genes and likely relies on activation of pre-existing cellular factors by viral proteins.

### Long chain acyl-CoA synthetases undergo limited proteolysis and changes in membrane association in infected cells, and Acsl3 is required to support effective poliovirus replication

**Figure 5:**
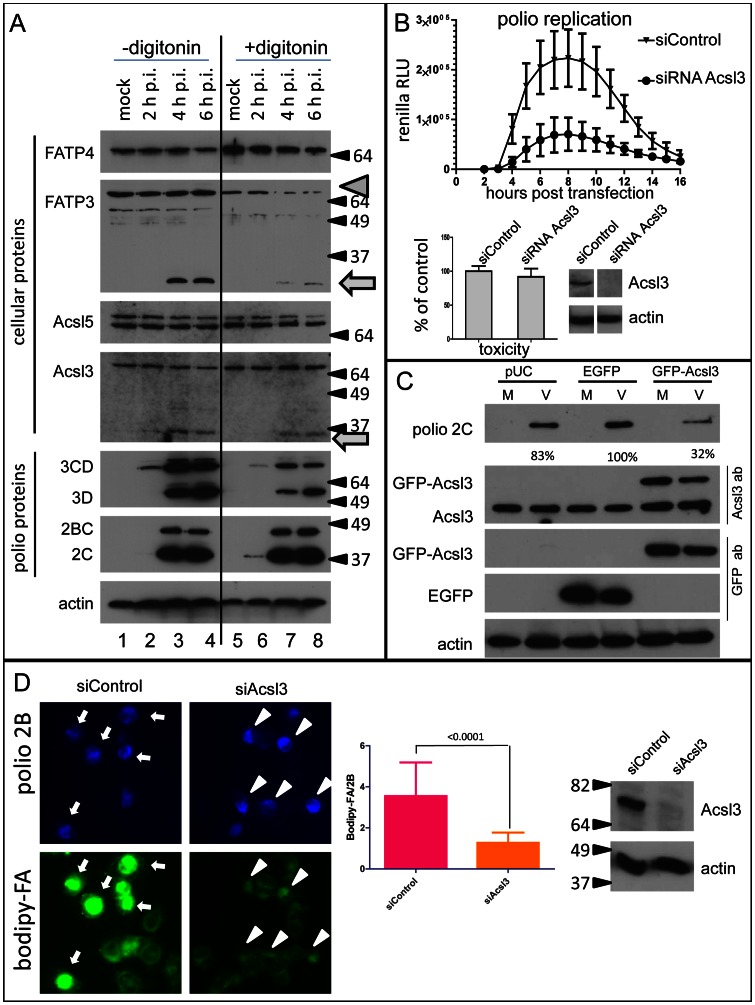
Cleavage and redistribution of long chain acyl-CoA synthetases in infected cells and requirement of functional Acsl3 for polio replication and FA import. **A**. HeLa cells infected at 50 PFU/cells were incubated for 2, 4, and 6 hours post infection and collected for Western blot after permeabilization with digitonin for 5 min at room temperature (lanes 5–8); control cells (lanes 1–4) underwent the same treatment but without the detergent. Proteins were detected by multiple western blots of the same membrane after stripping of previous antibodies. Actin is shown as loading control. Results from a representative experiment are shown. Arrows indicate cleavage products detected with anti-FATP3 and Acsl3 antibodies. Arrowhead points to the loss of FATP3 after digitonin treatment from infected cells. **B**. Acsl3 knock-down severely impairs polio replicon replication (top panel) while showing minimal cytotoxicity (lower panel). siRNA knock-down efficiency of Acsl3 protein is shown. **C**. Expression of a fusion protein GFP-Acsl3-HA reduces poliovirus replication. HeLa cells were transfected overnight with either empty pUC plasmid, pEGFP-N1 plasmid or pGFP-Acsl3-HA plasmid. Cells were infected (V) with poliovirus at 50 PFU/cell or mock-infected (M) and collected for analysis at 4 h p.i. Polio 2C band intensity is normalized to the EGFP expressing sample. Expression of GFP-Acsl3 protein is detected with either anti-Acsl3 antibodies (second panel) or anti-GFP antibodies (third panel) which also show expression of EGFP (forth panel). Actin is shown as loading control. **D**. Knock-down of ACSL3 expression reduces activation of FA import upon expression of poliovirus proteins. HeLa cells were transfected with control or ACSL-3-targeting siRNA and 48 h later they were transfected with the plasmid pTM-2A-3D coding for the entire poliovirus non-structural polyprotein fragment P2P3. The next day expression of polio proteins was induced by infection of cells with vaccinia-T7 virus. Bodipy-FA label was added for 30 min at 4 h post vaccinia-T7 infection. Statistical analysis of ∼150 cells from each sample shows bodipy-FA signal normalized to poliovirus antigen 2B fluorescence, p value is shown. Western blot shows ACSL3 knock-down, actin is shown as a loading control.

The human genome contains genes for 13 long and very long chain acyl-CoA synthetases that may facilitate FA uptake by the cells [22]. The data on expression profiles of these proteins as well as on their contribution to cellular metabolism are still very fragmentary and controversial [37].First, we monitored by western blot several long-chain acyl-CoA synthetases for which the reliable antibodies were available. We observed specific proteolytic cleavage of FATP3 and to a lesser extent Acsl3 proteins in infected cells, suggesting that their activity is being actively regulated (Figure 5A, arrows). Western blots of Acsl5 and FATP4 did not reveal obvious modifications of these enzymes in infected cells (Figure 5A). To see if association of acyl-CoA synthetases with cellular components is changed upon infection we treated the cells with digitonin. The membrane-targeted viral proteins 2C and 2BC were virtually totally recovered from the permeabilized cells. At the same soluble proteins 3D and 3CD were mostly lost upon cell permeabilization confirming optimal permeabilization conditions (Fig. 5A). Some amount of 3D and 3CD is expected to remain associated with the membrane-bound viral replication complexes. We observed significant loss of FATP3 protein from permeabilized cells at 4 and 6 h p.i. indicating that its association with cellular components is changing (Figure 5A, arrowhead). FATP3 was previously shown to be an-ER-localized protein with its N-terminus inserted into the ER lumen [21], and would be expected to remain in cells after digitonin treatment, as we see in the mock-infected cells. Thus its loss from the infected samples demonstrates that association of this protein with cellular structures is changing upon infection. Interestingly, FATP3 is one of the two long chain acyl-CoA synthetases undergoing proteolytic processing upon infection.

**Figure 6:**
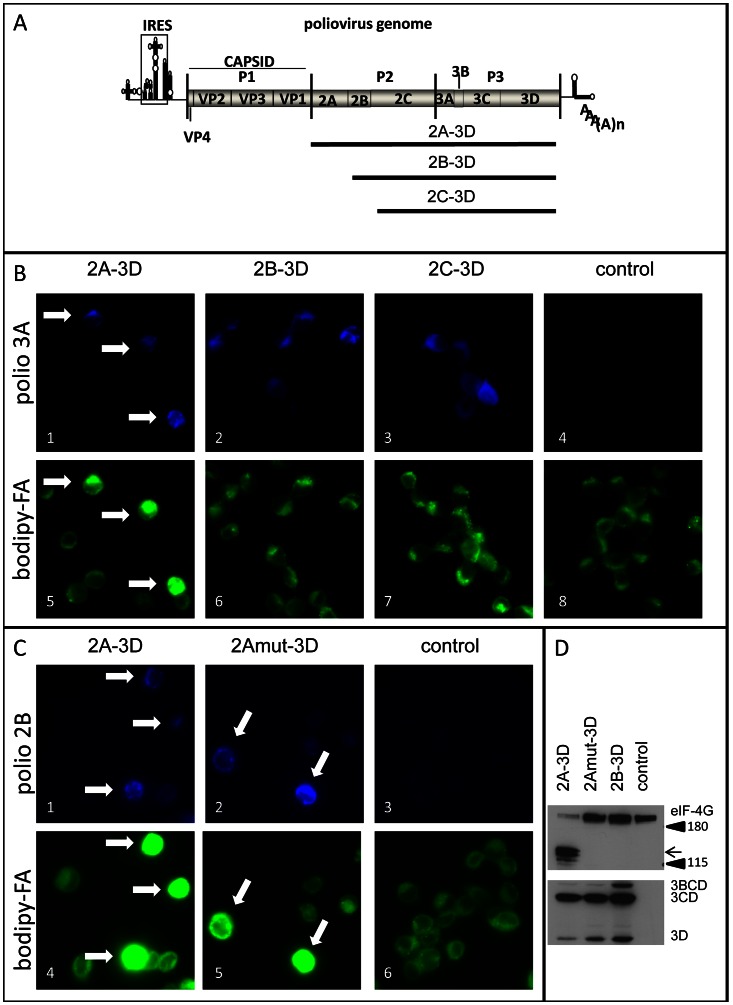
Activation of fatty acid import requires polio protein 2A. **A**. Schematic representation of poliovirus genome and truncated constructs used for expression of polio proteins. **B**. Only expression of the full P2P3 region activates import of bodipy-FA label (Arrows). HeLa cells were transfected with plasmids coding for the indicated polyprotein fragments under control of T7 promoter (empty vector for the control sample) The next day the cells were infected with vaccinia-T7 virus and labeled with bodipy-FA for 30 min at 4 h p. i. Poliovirus antigen 3A is detected as a marker of expression of viral polyprotein fragments 2A-3D (complete P2–P3), 2B-3D and 2C-3D. **C**. Protease activity of 2A is dispensable for activation of fatty acid import (arrows). HeLa cells were transfected with plasmids coding for the indicated polyprotein fragments under control of T7 promoter (empty vector for the control sample) The next day the cells were infected with vaccinia-T7 virus and labeled with bodipy-FA for 30 min at 4 h p. i. Poliovirus antigen 2B is detected as a marker of expression of the wt and 2A-mut containing P2–P3 polyprotein. **D**. Parallel samples to those shown in C were collected and analyzed for 2A protease activity and expression of viral proteins. Processing of eIF-4G (black arrow) is detected only in the sample expressing functional 2A protease (top panel). Accumulation of viral proteins detected by viral antigen 3D is comparable in all samples showing that the lack of 2A protease activity is because of the mutation, not because of the insufficient expression (lower panel).

To implement an unbiased approach to identify acyl-CoA synthetases that support replication of poliovirus we performed screen with siRNA pools targeting all 13 long chain acyl-CoA synthetases. Only siRNA against FATP5 showed significant toxicity in HeLa cells likely due to some non-specific effect (Figure S3) since this protein is believed to be expressed only in liver [38]. Depletion of other acyl-CoA synthetases was well tolerated by the cells, the apparent slight toxic effect rather reflects somewhat slower growth of cells treated with certain siRNA pools (Figure S3). Our initial screen identified three siRNA pools that induced significant, ∼80% reduction of replication: anti-acyl-CoA synthetase Bubblegum 2 (AcsBG2), FATP3 and Acsl3 (Figure S3). Western blot analysis of the targeted proteins revealed that only effect of Acsl3 siRNA was specific. AcsBG2 was not expressed in our HeLa cells as expected, since this protein was previously shown to be specific for brain stem and testis [39], and treatment of cells with either pooled or individual siRNAs against FATP3 did not result in significant reduction of the amount of the protein (not shown). All siRNAs from the anti-Acsl3 pool resulted in reduction of the targeted protein and decreased replication of PV, siRNA #2 was the most potent. The specificity of the ACSL3 knock-down effect was confirmed by rescue of polio replication by expression of the ACSL3 with mutated siRNA #2 targeting sequence (Figure S4). The strongest reduction of polio replication with the least cellular toxicity was observed after treatment of cells with the all four anti-Acsl3 siRNAs pool (Figure 5B). We also monitored PV infection in the cells expressing recombinant protein GFP-Acsl3-HA. Accumulation of viral proteins was significantly delayed in such cells, compared to cells expressing just EGFP, or transfected with an empty pUC plasmid (Fig. 5C), suggesting that fusion protein GFP-Acsl3-HA works like a dominant-negative mutant in the context of polio infection. Note that transfection efficiency of HeLa cells is about 60–80% and protein accumulation is measured in the total cell population, therefore the actual reduction of polio replication in transfected cells only should be even stronger. Since knock-down of Acsl3 expression inhibits polio replication, it is impossible to directly examine the role of Acsl3 in activation of FA import upon infection. Thus we expressed poliovirus non-structural P2P3 polyprotein fragment (Fig. 6A) in cells treated with control or ACSL3-targeting siRNAs with the help of vaccinia virus expressing T7 RNA polymerase [40]. This system is independent of polio replication and is discussed in details in the section below. Expression of poliovirus proteins was induced by infection of cells with vaccinia-T7 virus ∼72 hours post siRNA transfection. At 4 hours post vaccinia infection bodipy-FA probe was added to the media for 30 min. The cells treated with control siRNA which were positive for a polio antigen showed strong activation of FA import (Fig. 5D, arrows), while import of bodipy-FA in the cells with ACSL3 knock-down was significantly lower (Fig. 5E, arrowheads). The statistical analysis confirmed that bodipy-FA fluorescence normalized to polio protein 2B signal strongly declined in ACSL3 knockdown cells (Fig. 5D).

Our data show specific limited proteolysis of Acsl3 and FATP3 in infected cells, accompanied by the loss of membrane association of FATP3, which likely leads to modulation of activity of these enzymes, and demonstrate that functional Acsl3 is required for polio replication and is directly involved in import of FA upon expression of polio proteins.

### Poliovirus protein 2A is required but not sufficient for activation of FA import independent of its protease activity

To identify a PV protein(s) responsible for activation of FA import we expressed fragments of the viral polyprotein with the help of the vaccinia virus expressing T7 RNA polymerase [40]. The cells are transfected with a plasmid coding for a viral protein under control of T7 RNA polymerase promoter, and the next day they are infected with a vaccinia virus expressing T7 RNA polymerase gene. Thus the gene of interest is only expressed when T7 RNA polymerase accumulates in vaccinia-infected cells. This system provides rapid expression of high amount of recombinant proteins independent of nuclear transcription and RNA processing machinery, thus allowing synthesis of poliovirus proteins uncoupled from replication of viral RNA, on the timescale similar to the normal polio infection. It was successfully used previously to assess effects of individual PV proteins on cellular membrane architecture [41]. The P1 region of poliovirus genome codes for the structural proteins which are dispensable for replication, so we focused on the non-structural proteins encoded in the P2P3 genomic region (Figure 6A). The cells transfected with the plasmids coding for fragments of PV cDNA were infected with vaccinia virus, and bodipy-FA probe was added to the media for 30 min at 4 hours post vaccinia infection. All cells displayed significant vaccinia-induced CPE at that time (Fig. 6 B and C). Three of the polio proteins: 2B, 2C, 3A have membrane localization domains, and they have long been implicated in membrane rearrangements in infected cells [5], [6], [42]. However individual expression of 2B, 2C, 2BC, 3A, as well as 3CD and 3D did not result in increased FA import (not shown). Expression of the whole P2P3 polyprotein (2A-3D) (Fig. 6A) induced strong increase in bodipy-FA import (Fig. 6B 1 and 5, arrows; and Fig. S5). Expression of the 2B-3D or 2C-3D polyprotein fragments (Fig. 6A) never stimulated accumulation of bodipy-FA to the level comparable to the 2A-3D expressing cells (Fig. 6B 2, 6 and 3, 7; and Figure S5), showing that 2A is the protein responsible for triggering activation of FA import. Compared to the control cells infected with vaccinia-T7 virus after transfection with an empty vector, cells expressing 2B-3D fragment showed small, but reproducible increase in the baseline level of bodipy-FA accumulation (Figure S5). In the context of the P2P3 2A is expressed together with all the other non-structural poliovirus proteins and thus the activation of fatty acid import may depend on coordinated action of 2A and other viral factors. To investigate if expression of 2A alone can induce activation of FA import we generated a construct that expresses the 2A protein with an HA tag between amino-acids 144–145 since suitable anti-2A antibodies were not available. This position was previously identified to tolerate insertions in the context of the polio genome [43]. The full length polio RNA with 2A-HA had the same infectivity as the wt RNA, although it displayed somewhat smaller plaque phenotype (not shown), showing that 2A-HA is fully functional in the viral life cycle. When we expressed the 2A-HA protein individually it did not induce activation of FA import on its own (not shown). To investigate the requirement for 2A protease activity we engineered a point mutation in the 2A sequence substituting the catalytic amino acid C109 to A [44]. The lack of the protease activity of the 2A C109A mutant was confirmed by the absence of processing of eIF-4G, a well-established cellular target of 2A (Fig. 6D). Expression of the P2P3 piece of the PV polyprotein with the inactive 2A induced activation of the FA import like the wt construct (Fig. 6C), showing that complex role of 2A in modification of metabolism of infected cells is not restricted to the proteolitic processing of cellular proteins.

Thus poliovirus protein 2A is necessary for activation of FA import, independent of its protease activity, but expression of 2A alone is not sufficient and requires contribution from other viral non-structural proteins from the P2P3 region.

### Increase of FA import is a general phenomenon of picornavirus infection

**Figure 7:**
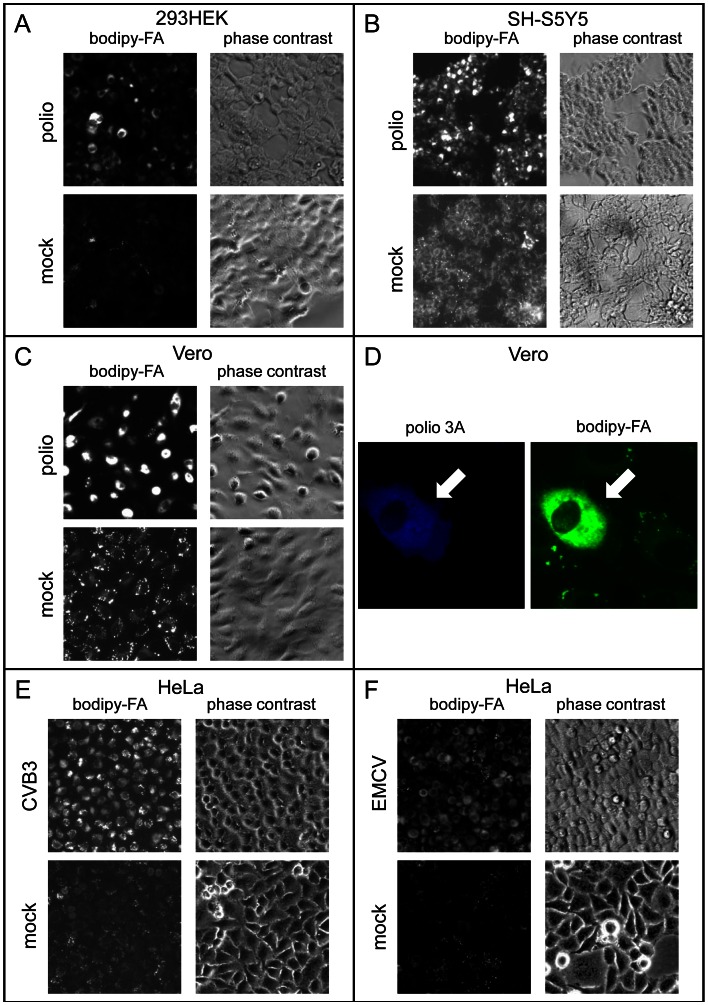
Activation of long chain fatty acid import is a general phenomenon of picornavirus infection. **A–C**. 293HEK (human embryonic kidney), SH-S5Y5 (human neuroblastoma) or Vero (green African kidney) cells were infected with poliovirus at 50 PFU/cell and incubated for 4 hours before 30 min label with bodipy-FA. Fluorescent and phase contrast images of infected and mock-infected cells are shown. **D**. Higher magnification of Vero cells infected with poliovirus and labeled with bodipy-FA like in C, showing that bodipy-FA accumulation is activated only in cells actively expressing viral proteins (arrow). **E, F**. HeLa cells were infected with either Coxsackie B3 virus or encephalomyocarditis virus at 50 PFU/cell and incubated for 4 hours before 30 min label with bodipy-FA. Fluorescent and phase contrast images of infected and mock-infected cells are shown.

Viruses in the animal host encounter diverse cellular environments, even when their tropism is limited to a few specific tissues. At the same time the core essential processes of replication machinery are expected to operate similarly in every cell type permissive for viral infection. To see if the activation of long chain FA import is a universal attribute of picornavirus infection, we assessed FA import in different types of cells upon infection with different picornaviruses. PV replication induced strong activation of bodipy-FA import in Vero (green African monkey kidney), 293HEK (human embryonic kidney) and SH-SY5Y (human neuroblastoma) cells similarly to what we observed previously in HeLa cells (Fig. 7A–D). The Figure 7B shows that only Vero cells actively expressing polio proteins demonstrate high FA import phenotype. To see if different viruses induce activation of FA import we compared PV-infected HeLa cells with the cells infected with Coxsackie virus B3 (CVB3), another enterovirus related to polio; as well as with a significantly more distantly related encephalomyocarditis virus (EMCV). These viruses efficiently replicate in HeLa cells with similar duration of their infection cycles (not shown). The cells infected with all these viruses showed strong activation of the bodipy-FA import which was distributed into similar membranous structures (Fig. 7 E–F).

These data show that activation of long chain FA import is a universal mechanism of altering host cell membrane metabolism activated by diverse picornaviruses in different cell types.

## Discussion

The generation of the membranous replication platforms is an indispensable step in the life cycle of all (+)RNA viruses of eukaryotes. The double membrane compartments observed at certain conditions in PV-infected cells prompted a hypothesis of the autophagy contribution to their formation which is also supported by the processing of the LC3 protein, a hallmark of autophagosome development, in infected cells [5], [45]. The available data also show that PV hijacks components of the cellular secretory pathway which carries coated vesicles between cellular organelles and plasma membrane. Rust et al. showed co-localization of PV protein 2B with the components of the COPII coat [6]. GBF1, a guanidine-nucleotide exchange factor for small GTPase Arf1 that coordinates formation of COPI-coated vesicles was shown to be a critical host factor for PV and CVB3 replication [46], [47]. Hsu et al. proposed that activation of Arf1 by GBF1 in infected cells results in recruitment of phosphoinositol-kinase 4 III β (PI4KIIIβ) instead of COPI coat. PI4KIIIβ generates PI4P lipid and diverts membranes from the secretory pathway to building viral membranous replication organelles [4].

However while the model of generation of viral replication membranes through subversion of the natural membrane remodeling machinery of the secretory cargo vesicles formation or autophagy seems logical and aesthetically appealing, it is difficult to reconcile with all the experimental data and it cannot explain membrane remodeling by even related viruses.

Picornaviruses show drastically different sensitivity to inhibition of the secretory pathway. PV is very sensitive to BFA, an inhibitor of GBF1 [48], [49], while EMCV or FMDV are totally refractory to the drug, and other picornaviruses demonstrate intermediate sensitivity to the inhibitor [7], [50]. It is likely that the components of the secretory pathway are necessary for the functionality of the poliovirus replication complexes, rather than for the development of the membranous scaffold of these structures. The characteristic membrane remodeling could be induced by expression of poliovirus proteins in the presence of BFA, showing that the functional GBF1-dependent pathways are not required to induce morphogenesis of the replication platforms [51].

Similarly picornavirus response to manipulation of autophagy varies greatly. Replication of human rhinovirus 2 was reported to be either dependent on induction of autophagy, or completely non-sensitive to manipulation of this pathway in different systems [8], [9].

Moreover, electron tomography studies show that replication organelles of PV and CVB3 represent complex tubular structures, rather than clusters of vesicles expected to be generated by vesicle-forming machinery of the secretory pathway or autophagy [52], [53].

The similar morphology of the replication structures of all picornaviruses strongly suggests that the mechanisms of their formation should be shared among different viruses [3], [54]. An intriguing clue comes from the old observations on the phospholipid synthesis in infected cells. Both poliovirus and EMCV were shown to strongly stimulate phospholipid synthesis [13], [55], while the former is sensitive BFA and the latter is completely resistant to the inhibitor [7], [48], [49].Our data presented here suggest that building of viral replication organelles to the large extent may rely on new membrane synthesis, unique to the infected cells, rather than on remodeling of pre-existing organelles through hijacking of membrane trafficking pathways. We demonstrate a rapid increase in long chain FA import into poliovirus-infected cells linked to activation of acyl-CoA synthetase activity. The overall cellular acyl-CoA synthetase activity was elevated as early as 2 hours post infection. Mock-infected cells largely incorporated FA in the lipid droplets, while in infected cells the imported FA was utilized mostly in highly activated PC synthesis, reflecting the rapid development of membranous replication platforms. The different substrate preference of acyl-CoA synthetase activity in infected cells translated into the overall significant perturbations in the composition of the PCs and increase of the diversity of PCs with shorter acyl chains. While we observed strong preference for import of C14 myristic acid in infected cells during the competition experiment, the cells preferentially accumulated C16/C18 PC species. This apparent controversy reflects the fact that during the competition assay the cells were incubated with bodipy-FA label and only one species of fatty acid was present in the media, while the cells assessed for changes in PC composition were incubated in standard media supplemented with serum. It is also possible that while myristic acid is being the preferred substrate for import into the infected cells, myristoyl-CoA may not be the preferred substrate for synthesis of PC. The actual composition of the pool of acyl-CoAs that eventually will be used for synthesis of new PC molecules is determined to a large extent by the availability of the corresponding FA substrates. Human serum for example may contain almost 30 times more palmitic (C16:0) and stearic (C18:0) acids than myristic acid [56].

The human genome contains 13 genes for acyl-CoA synthetases capable of activation of long and very long chain FAs (C12–C26) [22]. They have different expression profiles and distinct, although often overlapping substrate specificity, contributing to the complex tissue-specific regulation of the FA metabolism [23], [57]. siRNA knock-down and protein over-expression experiments show that polio replication requires functional Acsl3. Interestingly, both siRNA knock-down of Acsl3 and over-expression of a fusion protein GFP-Acsl3-HA was detrimental for poliovirus replication. It is possible that GFP and/or HA tags of the fusion protein specifically interfered with the Acsl3 function required to support the viral infection. It was reported that addition of the HA tag could significantly change cellular localization of a lipid droplet protein [30]. On the other hand, it is possible that the inhibition of polio replication resulted from the excess of Acsl3 activity in the cells over-expressing GFP-Acsl3-HA construct. For example it was shown that both depletion and over-expression of a chaperon protein DNAJC14 reduces replication of flaviviruses [58], suggesting that at least some cellular factors can support viral replication only at a narrow range of concentrations. We cannot exclude that other acyl-CoA synthetases are supporting viral replication. In this study we followed only the results of the siRNA screen that showed the most significant reduction of polio replication. It is possible that during the siRNA treatment targeting one acyl-CoA synthetase the cells would compensate this loss by increasing synthesis of other related proteins. Our siRNA data underscore that validation of the knock-down results on protein level is very important and that without it the popular high-throughput siRNA-based screens for host factors important for viral replication should be interpreted with caution.

The available data on expression, cellular localization and activity of long chain acyl-CoA synthetases is still fragmentary and often frustratingly contradictory. Acsl3 was reported to contribute to FA uptake by mammalian cells [20], [29]. In our experiments Acsl3 appeared to be directly involved in the activation of import of FA upon expression of polio proteins. Interestingly, rat Acsl3 preferentially activates short saturated FAs lauric (C12:0) and myristic (C14:0) in a biochemical assay [59] consistent with the strong effect of myristic acid in our competition experiments. Import of FAs into infected cells and polio replication were relatively insensitive to triacsin C (not shown), an inhibitor of rat acyl-CoA synthetases 1, 3, and 4 [60], [61]. However sensitivity of human and rat proteins to triacsin C may not be the same, moreover, as was reported for Acsl5, conflicting results could be observed in different assays for the same enzyme [62], [63]. It is also possible that sensitivity to the inhibitor may change upon interaction of Acsl3 with viral factors.

We also observed proteolytic cleavage of Acsl3 and FATP3 and loss of association of FATP3 with cellular structures upon infection, suggesting that modulation of acyl-CoA synthetase activity in infected cells is very specific. Poliovirus proteases 2A and 3C recognize YG or FG, and GQ bonds respectively, but the actual utilization of the cleavage sites depends on protein conformation and surrounding sequences [3]. Potential cleavage sites for the viral proteases that can generate the observed fragments are present in the Acsl3 and FATP3 sequences, but whether 2A or 3C actually directly cleave Acsl3 and/or FATP3 or if the proteolysis is performed by cellular proteases requires further investigation. The size of the small proteolytic products (∼30 KDa for FATP3 and ∼37 KDa for Acsl3) suggest that the cleavage site is located between the two conserved motifs characteristic of acyl-CoA synthetases [64], therefore the cleavage likely inactivates the enzymes. It should be noted that accumulation of the small cleavage products of FATP3 and Acsl3 was not accompanied by a significant decrease of the full length proteins, suggesting that proteolysis affects limited fractions of these proteins, possibly only in specific cellular locations which may help to redirect fatty acids from triglyceride synthesis pathway to production of PC.

Since poliovirus infection induces rapid shut-off of cellular cap-dependent mRNA translation and nuclear transcription [3], the most plausible mechanism of stimulation of FA import in infected cells is activation of pre-existing cellular acyl-CoA synthetases rather than the up-regulation of *de novo* expression of the enzymes. The lack of increase of the total amount of at least some acyl-CoA synthetases in infected cells, as well as resistance of stimulation of FA import to actinomycin D strongly support the idea of activation of pre-existing cellular factors upon infection.

Very little is known about the rapid posttranslational regulation of long chain acyl-CoA synthetases. It was shown that insulin may modulate acyl-CoA synthetase activity within rat adipocytes on the timescale of minutes, but the mechanism of this regulation is unknown [65].The fast activation of acyl-CoA synthetase activity in PV-infected cells required the viral protein 2A. 2A is responsible for the first cleavage of the polyprotein releasing the precursor of capsid proteins [3]. It also cleaves cellular proteins rendering cell environment favorable for viral replication. 2A-mediated cleavage of eIF4G results in inhibition of translation of cellular mRNAs and reorganization of translation apparatus to support IRES-driven translation of the PV RNA [66]. 2A proteases of PV and rhinoviruses can directly cleave nucleoporines leading to rapid disintegration of nucleo-cytoplasmic barrier in infected cells [67], [68]. The requirement of 2A for activating FA import independently of its protease activity represents a novel function of this protein in virus-cell interaction. Interestingly, previous studies showed that 2A has some role in polio replication unrelated to its protease function, but the nature of this contribution remained unclear [69]. Our data explain previous observations on expression of the PV proteins with known membrane-targeted sequences such as 2C and 2BC that failed to activate synthesis of new lipids in spite of producing complex membrane rearrangements [41], and shows that expression of the individual membrane-targeted proteins does not fully recapitulate complex modulation of membrane metabolism in infected cells.

Previous data show that replication of diverse (+)RNA viruses is intrinsically connected to the metabolism of long chain FAs. Replication of some picornaviruses as well as other (+)RNA viruses was shown to be sensitive to the inhibitors of the cellular FA synthase [70], [71], [72], [73]. Replication of brome mosaic virus in a yeast model depends on the activity of Delta-9 fatty acid desaturase [74], [75] and acyl-CoA binding protein Acb1p [76], consistent with the requirement for specific long chain FA for viral replication. Changes in lipid composition of the replication membranes compared to the membranes in non-infected cells was reported for diverse (+)RNA viruses [74], [77], supporting to the idea that viral replication complexes represent products of mostly *de novo* synthesis of new membranes with unique characteristics.

**Figure 8:**
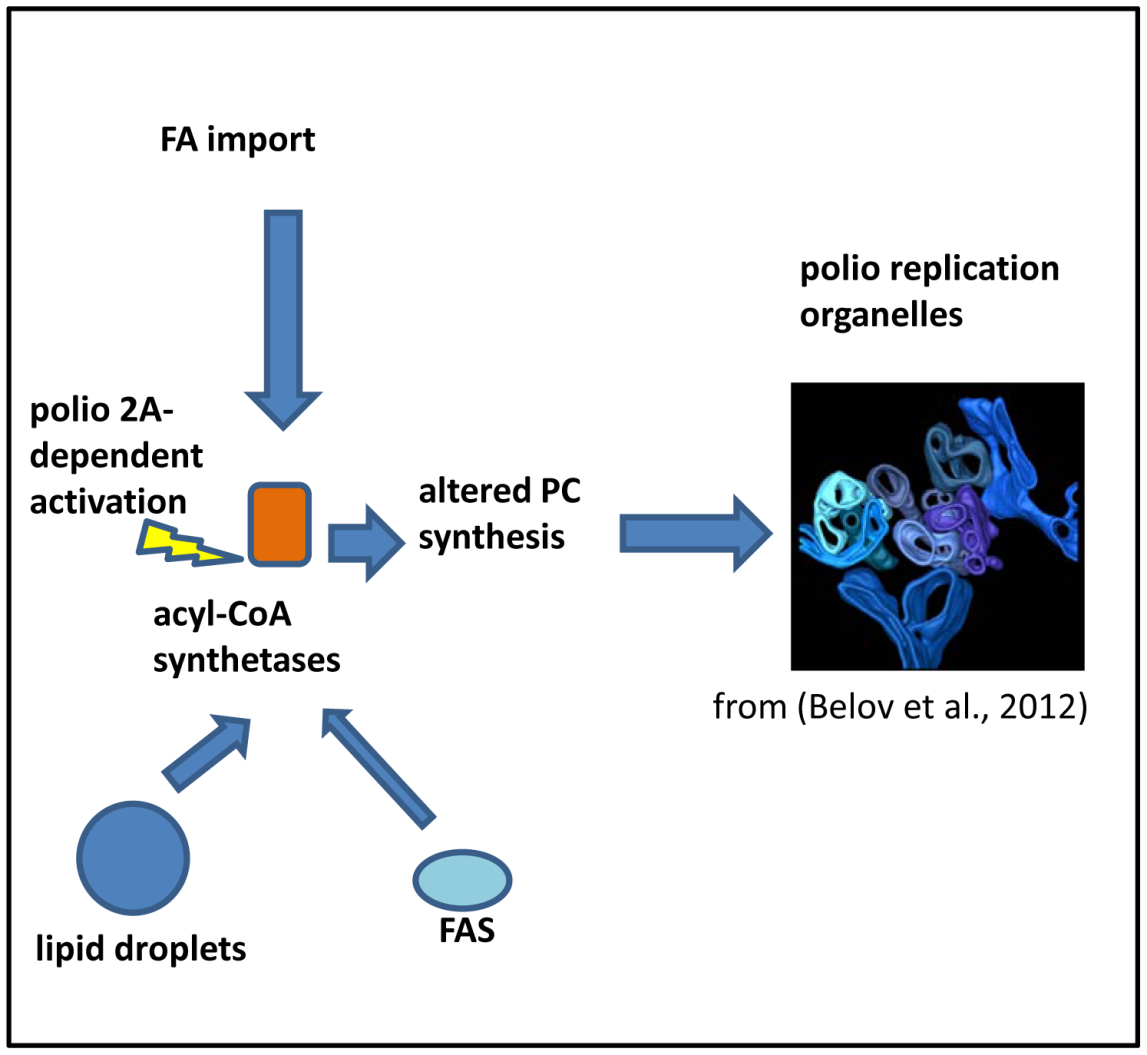
A model of the structural development of picornavirus replication complexes based on activation of cellular acyl-CoA synthetase activity by viral proteins (2A is required, but is not sufficient in case of polio). Elevated acyl-CoA synthetase activity leads to increased import of fatty acids (FA) form the media and also to utilization of FA form intracellular sources (FAS- fatty acid synthase)resulting in upregulated synthesis of altered species of phosphatidylcholines (PC). PCs with short fatty acid moieties preferentially synthesized in polio-infected cells spontaneously assemble into characteristic tightly packed convoluted tubular membranes structures constituting structural scaffold of poliovirus replication organelles.

Our data provide a foundation for a simple model that may explain the structural development of the membranous replication organelles shared by at least picorna-like viruses (Fig. 8). Certainly many aspects of this model are hypothetical at this point and require further investigations to elucidate the mechanistic details. The elevated acyl-CoA synthetase activity in infected cells inevitably increases import of FAs from the extracellular media but also would activate FAs released from intracellular sources, thus allowing utilization of resources in different cell types or growth conditions. The resulting excess of long chain acyl-CoAs would stimulate further steps in phospholipid synthesis and result in continuous extrusion of new membranes. Moreover the preference of acyl-CoA synthetase activity in infected cells for shorter FAs would result in generating membranes with higher fluidity with the intrinsic propensity to assemble into tight tubular structures (myelin figures) [78] surprisingly similar to the picornavirus replication membranes [52], [53]. These new membranes would need to be decorated with the necessary viral and cellular factors to make them capable of supporting viral replication, but the generation of the structural scaffold seems to be a unique process activated in infected cells, independent of the elements of the secretory pathway or autophagy. The distinct properties of the FA metabolism in infected cells and the widespread reliance of diverse (+)RNA viruses on their activation represent an attractive target for development of future broad spectrum antiviral therapeutics.

## Materials and Methods

### Cells and viruses

HeLa and 293HEK cells were maintained in DMEM medium supplemented with 10% heat-inactivated fetal bovine serum (FBS), Vero cells were grown in Eagle's MEM medium with heat-inactivated 10% FBS. Poliovirus type 1 Mahoney, Coxsackie B3 virus and encephalomyocarditis virus were propagated on HeLa cells and their titer was determined in a standard plaque assay. For experimental infections cell monolayer was washed once with serum-free DMEM, the virus was added according to the desired multiplicity of infection for 30 min at room temperature in serum-free DMEM buffered with 50 mM Hepes pH 7.4, after that the cells were incubated in standard or serum free media in standard growth conditions according to the experimental design.

### Plasmids

Plasmids pTM-2A-3D, pTM-2B-3D, pTM2C-3D coding for the corresponding fragments of poliovirus cDNA under transcriptional control of T7 promotor and translational control of EMCV IRES were a gift from Dr. Natalya Teterina (NIH). pXpA-RenR plasmid coding for polio replicon with *Renilla* luciferase gene substituting capsid region of polio genome was described elsewhere [79] Plasmid pTM-2Amut-3D containing mutation C109A in the 2A sequence was produced by point mutagenesis of the 2A sequence in pXpA-P2P3 [80] and recloning the PstI-SpeI fragment into pTM-2A-3D. For plasmid pXpA-SH-PV2A-HA coding for the full length polio cDNA containing 2A with HA tag the SpeI-SnaBI fragment containing 2A-HA was generated by two sequential overlapping PCRs and recloned into pXpA-SH [80]. Plasmid pTM-2A-HA for vaccinia-based expression of 2A protein with HA tag PmlI-SalI fragment containing part of EMCV IRES and the whole coding sequence for 2A-HA was synthesized by Life technologies and was cloned into pTM-1 vector (gift from Dr. Natalya Teterina, NIH). All polio constructs were verified by sequencing. Plasmids pGFP-ACSL3-HA and pcDNA3-ACSL3-HA were generously provided by Dr. Joachim Füllekrug, University of Heidelberg, Germany. Plasmid pCI-ACSL3 was made by cloning the PCR fragment coding for the wt ACSL3 sequence obtained from pcDNA3-ACSL3-HA into pCI expression vector (Promega). pCI-ACSL3^r^ was produced by mutating the anti-ACSL3 siRNA #2 targeting sequence (**bold**) GG**AAATGGATTACAGATGT**TG into GG**GAACGGCCTTCAAATGC**TG coding for the same amino acids GNGLQML. Plasmid HsCD00078974 coding for human ADRP protein (GenBank: BC005127.1) was purchased from DNASU plasmid collection (Arizona State University). Plasmid pmCherry-ADRP was constructed by cloning the PCR fragment coding for human ADRP into pmCherry-C1 vector (Clontech). All constructs were verified by sequencing.

### Vaccinia-T7 expression system

Purified recombinant vaccinia virus expressing T7 RNA polymerase (VT7-3 [40]) was a gift from Dr. Natalya Teterina, NIH. The day before experiment HeLa cells were transfected with pTM- or pXpA-based plasmids coding for fragments of polio cDNA under transcriptional control of T7 RNA polymerase promoter and translational control of EMCV or polio IRES respectively, with Mirus 2020 DNA transfection reagent according to manufacturer's protocol. The next day cells grown on glass cover-slips were infected with vaccinia VT7-3 virus at a multiplicity of 10 PFU/cell in serum-free DMEM for 1 hour at 37 C and then incubated for 4 more hours in standard growth media. After that the media was changed to pre-warmed serum-free media supplemented with 5 µM of fluorescent fatty acid analog Bodipy 500/510 C4–C9 and the cells were incubated for 30 more minutes, then washed once with PBS and fixed with 4% formaldehyde in PBS and processed for microscopy analysis.

### Antibodies

Anti-Acsl3 mouse polyclonal antibodies were from Abnova; anti-Acsl5 mouse monoclonal antibodies were from Abcam; rabbit polyclonal anti-FATP3, anti-FATP4, anti-Acs bubblegum 2 were described in [81], [82], [83]. Mouse monoclonal anti-polio 2B and 3A antibodies were a gift from Prof. K. Bienz, University of Basel, Switzerland. Rabbit polyclonal anti-polio 3D antibodies were made by Chemicon using recombinant polio 3D protein as immunogen. Secondary anti-mouse and anti-rabbit highly cross-adsorbed goat antibodies conjugates with Alexa 350, Alexa 498 and Alexa 595 were from Molecular Probes.

### Reagents

Fluorescent fatty acid Bodipy 500/510 C4–C9 (bodipy-FA) was from Molecular probes. Unlabeled long chain fatty acids, Coenzyme A (CoA), α-cyclodextrin, ATP and neutral and phospholipid standards were from Sigma-Aldrich. Phosphatidylinositol 4 phosphate from porcine brain was from Avanti Polar Lipids. Fatty acid stock solutions were prepared in DMSO. Neutral and phospholipids were dissolved in chloroform.

### siRNAs

siGenome siRNA pools and corresponding individual siRNA oligos targeting human long chain acyl-CoA synthetases were from Dharmacon. HeLa cells were plated at 10000 cells/well in a 96 well plate and transfected with siRNA with Dharmafect 1 transfection reagent (Dharmacon) according to manufacturer's recommendations. After 72 hours of incubation with siRNA the cells were used for polio replicon replication assay. Toxicity of siRNA treatment was assessed with Cell-Titer Glo luminescent assay (Promega).

### Polio replicon replication assay

Polio replicon assay was performed as described in [51] with minor modifications. Briefly, HeLa cells grown on 96-well plates were transfected with polio replicon RNA with mRNA trans-It kit (Mirus) and incubated in TECAN Infinite M1000 plate reader at 37 C for 16 hours in standard growth media supplemented with 30 µM live cell Renilla substrate EnduRen (Promega). Measurements of Renilla luciferase activity were taken every hour post transfection, and the signal for each sample is averaged from at least 8 wells of the 96 well plate.

### Microscopy

For immunofluorescent microscopy cells were fixed with 4% formaldehyde in PBS for 20 min. The cells labeled with Bodipy 500/510 C4–C9 were incubated with primary and secondary antibodies in 0.02% saponin in PBS with 5% FBS for 1 hour and were washed 3 times with PBS after each incubation. Secondary antibodies conjugated to Alexa 350 were used for detection of viral antigens in Bodipy 500/510 C4–C9-labeled cells since we found that Bodipy 500/510 emits strong fluorescence in both green and red spectra after detergent treatment required for immunodetection of proteins, apparently due to the red shift in fluorescent spectrum of Bodipy when the fluorescent groups come in close contacts (Molecular Probes manual). Without detergent permeabilization red fluorescence of Bodipy-labeled lipids was negligible, thus allowing use of pmCherry-fused ADRP protein for detection of lipid droplets. Images were taken with Zeiss Axiovert 200M fluorescent microscope equipped with Axiocam Mrm monochrome digital camera; confocal images were obtained with a Zeiss ApoTome AxioImager M2 microscope.

### Image processing

Colors were artificially assigned to black and white microscope images using Adobe Photoshop software according to the imaging channel. All software processing was applied universally to the whole image and images from different samples were taken and processed using identical conditions. Fluorescence intensity in individual cells was evaluated with ImageJ software (NIH). Co-localization pattern was visualized with ImageJ software (NIH) co-localization module by Pierre Bourdoncle, Institut Jacques Monod, Service Imagerie, Paris.

### Digitonin permeabilization assay

HeLa cells grown on 12-well plate were infected with poliovirus and incubated for 4 hours in standard growth conditions. Permeabilization was performed at room temperature. The cells were washed once with KHM buffer (110 mM K-acetate, 2 mM MgCl_2_, 20 mM HEPES-KOH, pH 7.4) and incubated for 5 min in 50 µg/ml fresh digitonin solution in KHM (KHM buffer without digitonin for control cells). After that the cells were washed twice with KHM and lysed with mild lysis buffer (0.1M Tris-HCl pH 7.8; 0.5% Triton-×100) supplemented with protease inhibitors cocktail (Sigma-Aldrich). The lysate cleared by low-speed centrifugation was used for western blot analysis.

### Western blot membrane stripping

Multiple western blots were performed after stripping the membrane with Re-Blot Plus solutions (Chemicon) according to manufacturer's recommendations. Western blots were developed with ECL prime or ECL select chemiluminescence kits (GE Healthcare).

### Fatty acid import assay

For fatty acid import assay infected or mock-infected cells were incubated in standard or serum-free media depending on experiment design. After indicated time post infection the media was replaced for new pre-warmed media with 0.4 µM Bodipy 500/510 C4–C9 and after 30 min incubation the cells were fixed with 4%formaldehyde in PBS for 20 min, washed with PBS and used for microscope imaging and/or fluorescence reading on TECAN Infinite M1000 plate reader.

### FACS analysis of bodipy-FA import

Cells labeled with bodipy-FA as described above were detached from the plate with versen solution, fixed for 1 h with 1% paraformaldehyde in PBS and analyzed with FACSAriaII cell sorter (BD). The total of 10000 events were used for fluorescence analysis with FloJo software after gating in FCS and SSC mode for single cell population.

### Fatty acid import competition assay

HeLa cells were grown on a 96 well tissue culture plate with transparent bottom overnight and the next day infected with poliovirus at 50 PFU/cell. After infection the cells were incubated either in standard or serum-free media as indicated. At 4 h p. i. the media was replaced with the same type of pre-warmed media supplemented with 0.4 µM of bodipy-FA label, 50 µM of the competitor fatty acid and 1 µg/ml of cell-permeable DNA stain Hoechst 33342. After 30 min incubation the cells were washed with PBS and fixed with 4% formaldehyde in PBS and the Hoechst and bodipy-FA fluorescence were read in TECAN Infinite M1000 plate reader at excitation/emission 340/455 and 490/520 respectively. The bodipy-FA signal was normalized to Hoechst to account for variability in cell density and the data for each fatty acid are averaged form 12 wells. The data are expressed as percentage of the signal from control mock-infected cells incubated without any competitor fatty acid. Statistical analysis was performed with GraphPad PRIZM software.

### Acyl-CoA synthetase activity measurement

HeLa cells grown on T75 flasks were harvested, washed 3 times with cold PBS and re-suspended in STE buffer (8.5% sucrose, 10 mM Tris-HCl pH 8. 0.5 mM EDTA) supplemented with protease inhibitors cocktail (Sigma-Aldrich). Cells were lysed by twice freeze-thawing and the protein concentration of the lysates was determined by Bradford method. The assay mix containing 500 nM Bodipy 500/510 C4–C9 substrate solubilized with α-cyclodextrin (10 mg/ml in 10 mMTris-HCI pH 8.0), 40 mM Tris-HCI pH 7.5, 10 mM ATP, 10 mM MgCl_2_, 0.2 mM CoA, 0.2 mM dithiothreitol was assembled on ice, and the reaction was started by addition of an aliquot of cell suspension containing 60 µg of total protein. Duplicate reactions were incubated at 37 C for 20 min and terminated by the addition of Dole's solution (isopropanol∶heptane∶2N H_2_SO_4_ 40∶10∶1). Newly synthesized fluorescent acyl-CoA was recovered in aqueous phase after 4 extractions with heptane and the fluorescence was measured by TECAN Infinite M1000 plate reader. Statistical analysis was performed with GraphPad PRIZM software.

### Lipid extraction

Lipid extraction was performed according to Folch method [84]. Cells grown on T75 flasks were harvested, resuspended in STE buffer (8.5% sucrose, 10 mM Tris-HCl pH 8. 0.5 mM EDTA) and the protein concentration of cell suspension was determined by Bradford method. An aliquot of cell suspension containing 1500 µg protein was adjusted to 250 µl by STE in a glass tube and 3.75 ml of chloroform∶methanol (2∶1) with 5 mM HCl mix was added. The tube was vortexed for 30″, then 0.75 ml H_2_O was added, and the tube was vortexed again for 30″ and centrifuged at 1500 rpm in a tabletop centrifuge for 5 min. The top aqueous phase was discarded and lower organic phase was extracted 3 more times with Folch theoretical upper phase (chloroform∶methanol∶H_2_O 3∶48∶47). After extractions lipid-containing organic phase was dried under a stream of nitrogen gas. Lipids were re-suspended in 50 µl of chloroform before loading on TLC plates.

### Thin-layer chromatography (TLC)

Thin layer chromatography glass silica plates (Analtech) were prewashed with chloroform∶methanol (1∶1) and air-dried. Polar lipids were separated in chloroform∶ethanol∶water∶triethylamine (30∶35∶7∶35) and neutral lipids were separated in hexane∶ether∶acetic acid (80∶20∶1). Phospholipids were stained with Phospray (Sigma Aldrich) and neutral lipids were detected with bromothymol blue spray (Sigma Aldrich).

### Lipid analysis by TLC-MALDI

Total lipid extracts of infected HeLa cultures were reconstituted in equal volumes 2∶1 chloroform∶methanol (v∶v). 20% of the reconstituted lipids were spotted onto aluminum-backed silica gel 60 F_254_ TLC plates (Merck, Darmstadt, Germany). TLC plates were developed in an equilibrated chamber of 65∶25∶4 chloroform∶methanol∶ammonium hydroxide (v∶v∶v), then removed and dried under a gentle nitrogen stream. Plates were prepared for MALDI by spray-coating with at least 5 mL of a 20 mg/mL solution of Norharmane (9H-Pyrido[3, 4-b]indole) in 2∶1 chloroform∶methanol (v∶v). Plates were dried under a gentle nitrogen stream and mounted to a TLC-MALDI adapter. TLC-MALDI software within Compass 1.3 was used to define lane length, width, raster distance, and total shots (300 per position, 900 shots summed from 3 x-step, width, 1 y-step per millimeter, height). Plates were read in reflectron-positive mode with a mass window of 660–1060 *m*/*z*. Peak intensity values were used to summate PC intensity. These intensities were transformed to ratios of PC subset/PC total. To accommodate for lane-to-lane variation these ratios were normalized to mock infected ratios, represented as a percentage change.

To evaluate changes in the diversity of PC species during infection the total PC spot was deconstructed by Rf value and split into 2 groups, low Rf PC and high Rf PC. For each group the number of unique *m*/*z* values detected were tallied within an experimental group. Unique low or high values are represented as a percentage over total PC diversity. These unique *m*/*z* values likely represent acyl chain unsaturation patterns, whose abundance, detection threshold, and exact identity cannot be adequately confirmed with this technique. All reagents were obtained through Sigma-Aldrich (St. Louis, MO) unless otherwise noted. Commercial phosphatidylcholine (PC) standards were obtained from Avanti Polar Lipids, Inc. (Alabaster, Alabama) containing different acyl chain configurations. These PC standards were used to define TLC migration in the respective solvent system and used as mass standards for MALDI identification, 2 carbon unit fatty acid changes are easily identified first by *m/z* 28 unit changes and secondarily by subtle changes in migration. Autoflex Speed MALDI-TOF/TOF-MS, ImagePrep, and MS-related software were sourced from Bruker Daltonics (Billerica, MA).

## Supporting Information

Figure S1 HeLa cells were infected with poliovirus at 50 PFU/cell and incubated in media with or without serum. Bodipy-FA label was added for 30 min at 4 h p. i. Lipids were extracted and resolved by thin layer chromatography. Neutral lipids resolved in hexane∶ether∶acetic acid (80∶20∶1) system. Polar lipids resolved in chloroform∶ethanol∶water∶triethylamine (30∶35∶7∶35) system. FFA-lipids represent fluorescent fatty acid-containing lipids synthesized during 30 min labeling period. Total neutral lipids were stained with bromothymol blue. Total phospholipids were stained with Phostain. **Samples:** **1**. Lipids from infected cells incubated with serum. **2**. Lipids from mock-infected cells incubated with serum. **3**. Lipids from infected cells incubated without serum. **4**. lipids from mock-infected cells incubated without serum. **Markers:** **5**. Stearic acid (C18:0). **6**. Palmitic acid (C16:0). **7**. Linoleic acid (C18:2). **8**. Free Bodipy 500/510 C4–C9 9 (bodipy-FA). **Neutral lipid markers:** **9**. Cholesteryl palmitate (esterified cholesterol). **10**. Cholesterol. **11**. 1-monostearoyl-rac-glycerol (monoglyceride). **12**. 1,2-dipalmitoyl-rac-glycerol (diglyceride). **13**. 1,3-Dipalmitoyl-2-oleoylglycerol (triglyceride). **Polar lipid markers:** 9*. L-a-Phosphatidyinositol-4-phosphate. 10*. 1,2-Dipalmitoyl-sn-glycero-3-phosphate (phosphatidic acid). 11*. 3-sn-phosphatidyl-l-serine. 12*. 1,2-Dipalmitoyl-glycero-3-phosphoethanolamine (phosphatidylethanolamine). 13*. 2-Oleoyl-1-palmitoyl-sn-glycero-3-phosphocholine (phosphatidylcholine).
(PDF)

Figure S2 **A**. Effect of the inhibitors on polio replicon replication. HeLa cells grown on a 96 well plate were transfected with a polio replicon RNA with the Renilla luciferase gene substituting capsid region. Luminescence was monitored in live cells incubated with Endu-Ren substrate added in the media. Actinomycin D (**AMD**, an inhibitor of nuclear transcription) was added to the cells for 30 min before the replicon transfection and was present in the incubation media thereafter at 5 µg/ml. Cycloheximide (**CHI**, an inhibitor of mRNA translation) and Guanidine-HCl (**Gua** a specific inhibitor of polio replication at this concentration) were added at the time of transfection at 10 µg/ml and at 2 mM respectively. Equivalent amount of DMSO (solvent for AMD) and water (solvent for CHI and Gua) were added to the control cells. **B**. and **C**. HeLa cells were pre-incubated for 30 min with 5 µg/ml AMD) (equivalent amount of the DMSO solvent was added to the control cells) and infected with poliovirus at 50 PFU/cell. After 4 hours incubation in the standard media in the presence of AMD (DMSO in control) the cells were labeled for 30 min with 0.4 µM bodipy-FA in pre-warmed serum-free media also in the presence of the inhibitor (DMSO in control). After the incubation with the label the cells were washed with PBS and fixed with 4% formaldehyde in PBS. Fluorescence (bodipy-FA) and phase contrast images are shown. **D–F**. HeLa cells were infected with poliovirus at 50 PFU/cell and incubated in standard growth media for 3.5 h; after that the media was replaced with pre-warmed media containing: **D**. (control) equivalent amount of water (solvent for CHI and Gua) **E**. 10 µg/ml CHI. **F**. 2 mM GUA. The cells were incubated for 30 more min, and then the media was replaced with pre-warmed serum-free media containing the same inhibitors (water in control) and supplemented with 0.4 µM of bodipy-FA label. After 30 min incubation with the label the cells were washed with PBS and fixed with 4% formaldehyde in PBS. Fluorescence (bodipy-FA) and phase contrast images are shown.
(PDF)

Figure S3 HeLa cells grown on 96 well plates were transfected with siGenome siRNA pools targeting all known human long and very long chain acyl-CoA synthetases, 16 wells for each siRNA pool. siControl scrambled siRNA (Dharmacon) served as a control. After 72 h incubation with siRNA polio replicon replication assay was performed. Total replication is calculated as area under curve using Prizm software and the data are displayed as percentage of control. Toxicity was measured after replicon replication assay. siRNAs exhibited the strongest effect on replication (Acsl3, Acs BG2 and FATP3) are outlined in blue boxes. The toxic FATP5 siRNA is outlined by the red box.
(PDF)

Figure S4 HeLa cells grown on 96 well plates were transfected with each of four individual siRNAs from the siGenome pool targeting long chain acyl-CoA synthetase 3 (Acsl3), 16 wells for each siRNA. siControl scrambled siRNA (Dharmacon) served as a control. After 72 h incubation with siRNA polio replicon replication assay was performed. Total replication is calculated as area under curve using Prizm software and the data are displayed as percentage of control. Toxicity and western blot analysis were performed after replicon replication assay. For the siRNA rescue experiment cells were transfected with the most potent anti-Acsl3 siRNA #2 (or control siRNA) and in ∼48 hours they were transfected with pCI-Acsl3^r^ plasmid coding for the Acsl3 sequence with mutated siRNA targeting site (control samples were transfected with an empty vector). The next day after DNA transfection polio replicon assay was performed.
(PDF)

Figure S5 HeLa cells were transfected with plasmids coding for the indicated polyprotein fragments under control of T7 promoter (empty vector for the control sample). The next day the cells were infected with vaccinia-T7 virus and labeled with bodipy-FA for 30 min at 4 h p. i. (one control sample was incubated without bodipy-FA to measure the background cell fluorescence). After that the cells were collected, fixed in 1% PFA and processed for FACS analysis (10000 cells per sample). Aliquots of the corresponding samples were analyzed for 2A protease activity (middle panel) and expression of the viral proteins (lower panel). Processing of eIF-4G (black arrow) is detected only in the sample expressing functional 2A protease. Asterisks (*) indicate background bands showing equal amount of the loaded material.
(PDF)
