# Supplementary material for: Increased Long Chain acyl-Coa Synthetase Activity and Fatty Acid Import Is Linked to Membrane Synthesis for Development of Picornavirus Replication Organelles
Source: PLoS Pathog. 2013 Jun 6;9(6):e1003401. doi: 10.1371/journal.ppat.1003401 (PMC3675155; doi:10.1371/journal.ppat.1003401)
Supplement: Figure S1 — HeLa cells were infected with poliovirus at 50 PFU/cell and incubated in media with or without serum. Bodipy-FA label was added for 30 min at 4 h p. i. Lipids were extracted and resolved by thin layer chromatography. Neutral lipids resolved in hexane∶ether∶acetic acid (80∶20∶1) system. Polar lipids resolved in chloroform∶ethanol∶water∶triethylamine (30∶35∶7∶35) system. FFA-lipids represent fluorescent fatty acid-containing lipids synthesized during 30 min labeling period. Total neutral lipids were stained with bromothymol blue. Total phospholipids were stained with Phostain. Samples: 1. Lipids from infected cells incubated with serum. 2. Lipids from mock-infected cells incubated with serum. 3. Lipids from infected cells incubated without serum. 4. lipids from mock-infected cells incubated without serum. Markers: 5. Stearic acid (C18:0). 6. Palmitic acid (C16:0). 7. Linoleic acid (C18:2). 8. Free Bodipy 500/510 C4–C9 9 (bodipy-FA). Neutral lipid markers: 9. Cholesteryl palmitate (esterified cholesterol). 10. Cholesterol. 11. 1-monostearoyl-rac-glycerol (monoglyceride). 12. 1,2-dipalmitoyl-rac-glycerol (diglyceride). 13. 1,3-Dipalmitoyl-2-oleoylglycerol (triglyceride). Polar lipid markers: 9*. L-a-Phosphatidyinositol-4-phosphate. 10*. 1,2-Dipalmitoyl-sn-glycero-3-phosphate (phosphatidic acid). 11*. 3-sn-phosphatidyl-l-serine. 12*. 1,2-Dipalmitoyl-glycero-3-phosphoethanolamine (phosphatidylethanolamine). 13*. 2-Oleoyl-1-palmitoyl-sn-glycero-3-phosphocholine (phosphatidylcholine). (PDF) [file ppat.1003401.s001.pdf]

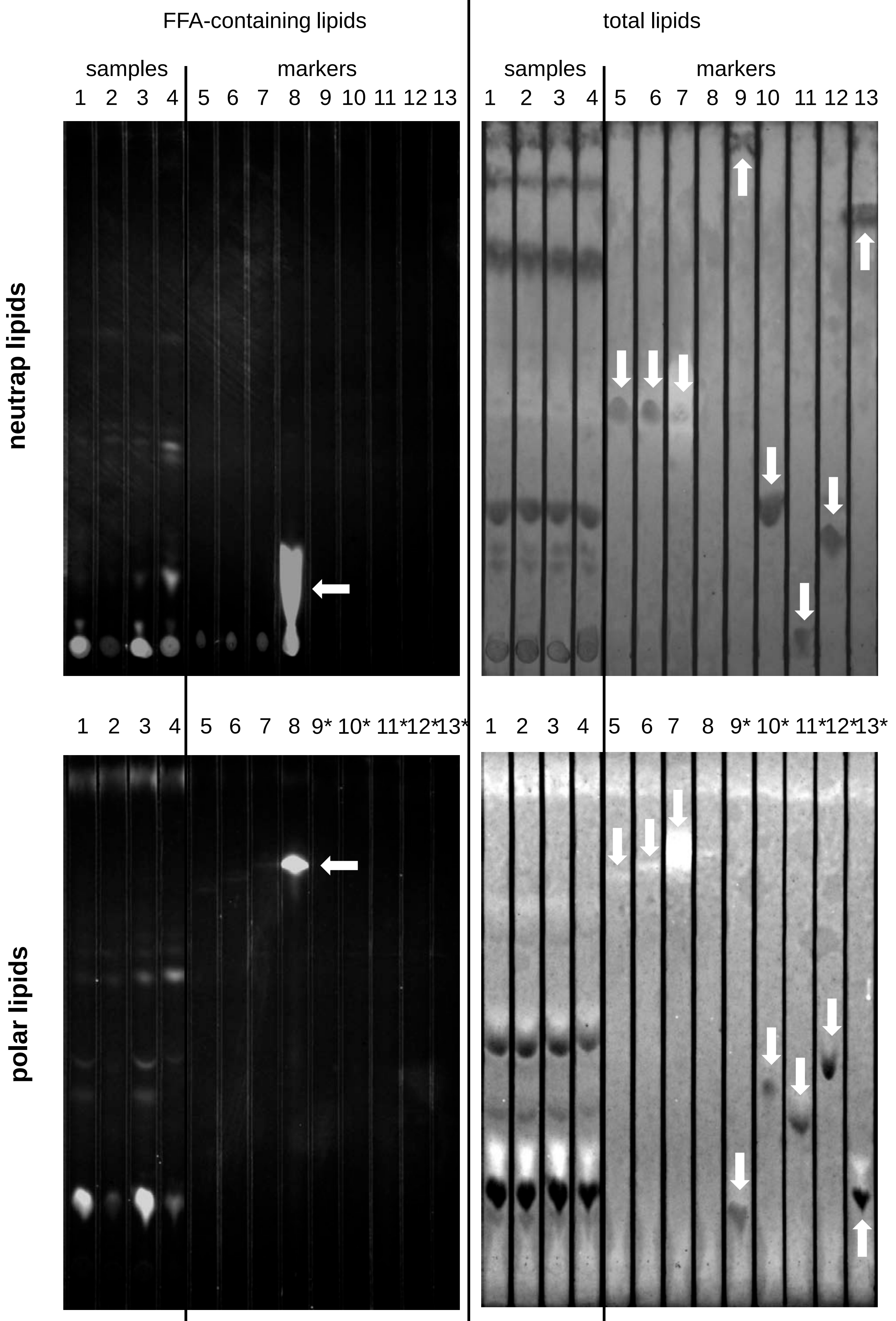
FFA-containing lipids
total lipids
samples
markers
1 2 3 4 5 6 7 8 9 10 11 12 13
neutrap lipids
polar lipids
1 2 3 4 5 6 7 8 9* 10* 11* 12* 13*
