# Supplementary figures and images for: Increased Long Chain acyl-Coa Synthetase Activity and Fatty Acid Import Is Linked to Membrane Synthesis for Development of Picornavirus Replication Organelles
Source: PLoS Pathog. 2013 Jun 6;9(6):e1003401. doi: 10.1371/journal.ppat.1003401 (PMC3675155; doi:10.1371/journal.ppat.1003401)

**A**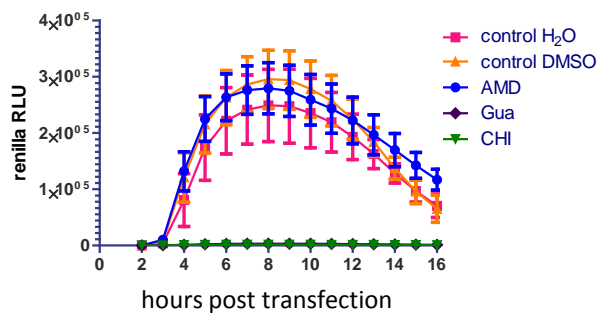**B**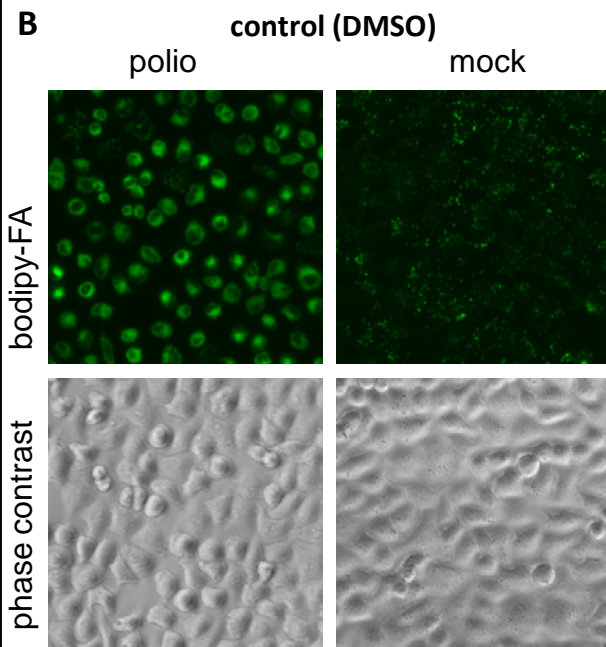**C**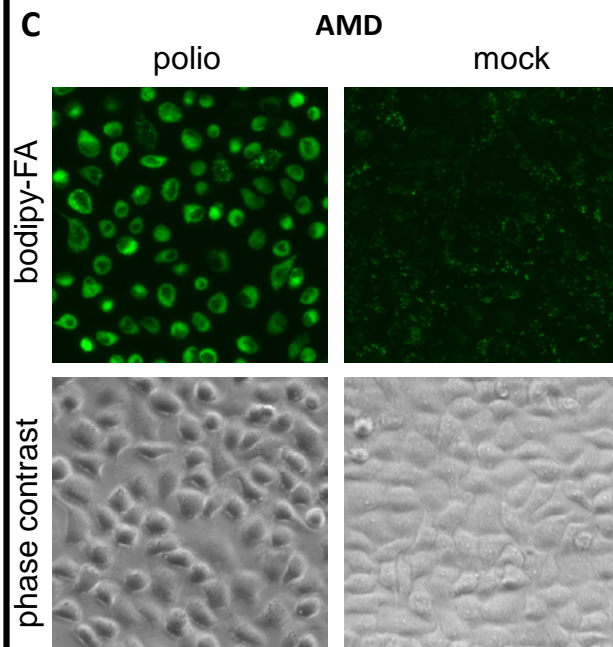**D**

**control (H<sub>2</sub>O)**

polio      mock

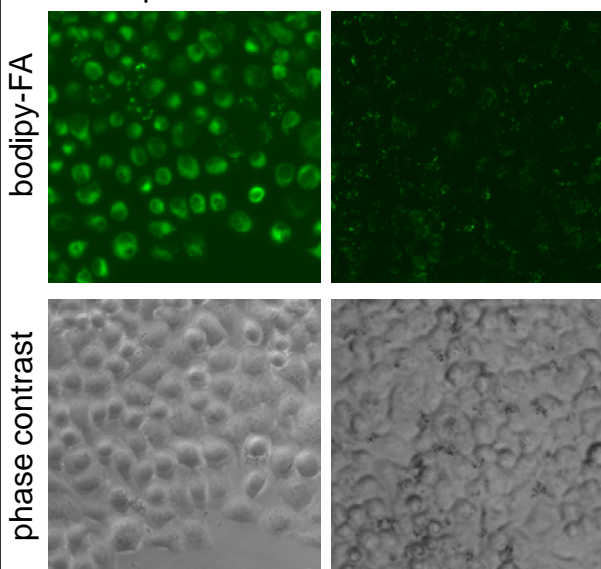**E**

**GUA**

polio      mock

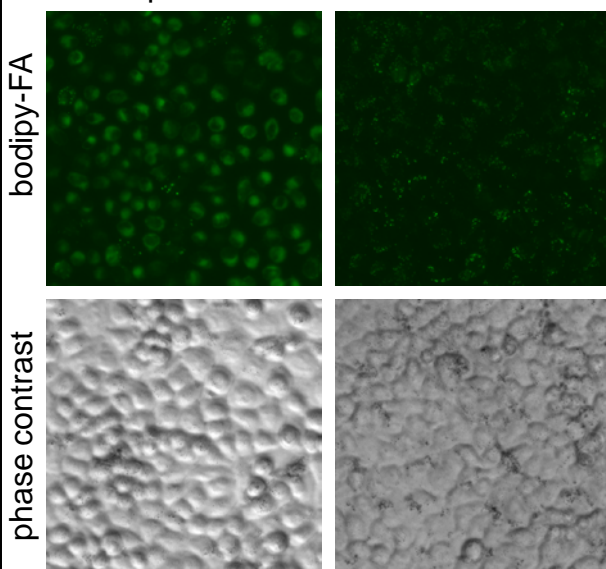**F**

**CHI**

polio      mock

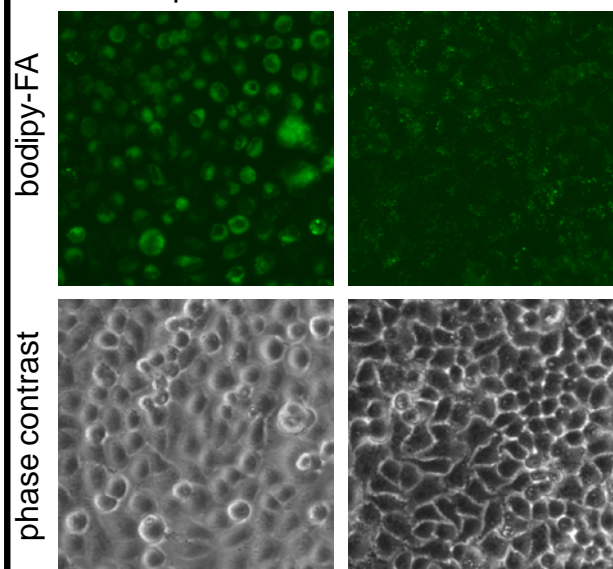

Supplement: Figure S2 — A. Effect of the inhibitors on polio replicon replication. HeLa cells grown on a 96 well plate were transfected with a polio replicon RNA with the Renilla luciferase gene substituting capsid region. Luminescence was monitored in live cells incubated with Endu-Ren substrate added in the media. Actinomycin D (AMD, an inhibitor of nuclear transcription) was added to the cells for 30 min before the replicon transfection and was present in the incubation media thereafter at 5 µg/ml. Cycloheximide (CHI, an inhibitor of mRNA translation) and Guanidine-HCl (Gua a specific inhibitor of polio replication at this concentration) were added at the time of transfection at 10 µg/ml and at 2 mM respectively. Equivalent amount of DMSO (solvent for AMD) and water (solvent for CHI and Gua) were added to the control cells. B. and C. HeLa cells were pre-incubated for 30 min with 5 µg/ml AMD) (equivalent amount of the DMSO solvent was added to the control cells) and infected with poliovirus at 50 PFU/cell. After 4 hours incubation in the standard media in the presence of AMD (DMSO in control) the cells were labeled for 30 min with 0.4 µM bodipy-FA in pre-warmed serum-free media also in the presence of the inhibitor (DMSO in control). After the incubation with the label the cells were washed with PBS and fixed with 4% formaldehyde in PBS. Fluorescence (bodipy-FA) and phase contrast images are shown. D–F. HeLa cells were infected with poliovirus at 50 PFU/cell and incubated in standard growth media for 3.5 h; after that the media was replaced with pre-warmed media containing: D. (control) equivalent amount of water (solvent for CHI and Gua) E. 10 µg/ml CHI. F. 2 mM GUA. The cells were incubated for 30 more min, and then the media was replaced with pre-warmed serum-free media containing the same inhibitors (water in control) and supplemented with 0.4 µM of bodipy-FA label. After 30 min incubation with the label the cells were washed with PBS and fixed with 4% formaldehyde in PBS. Fluorescen [file ppat.1003401.s002.pdf]

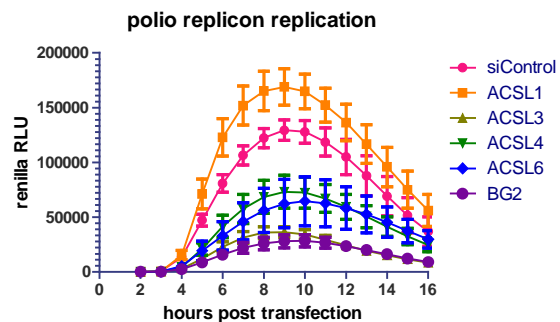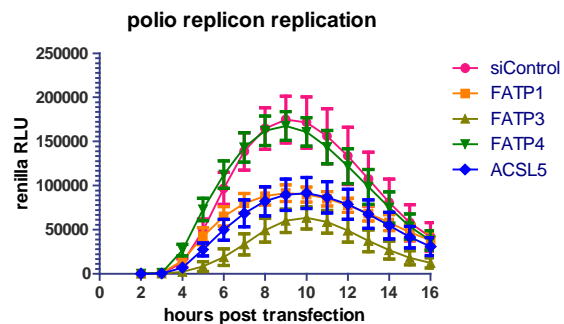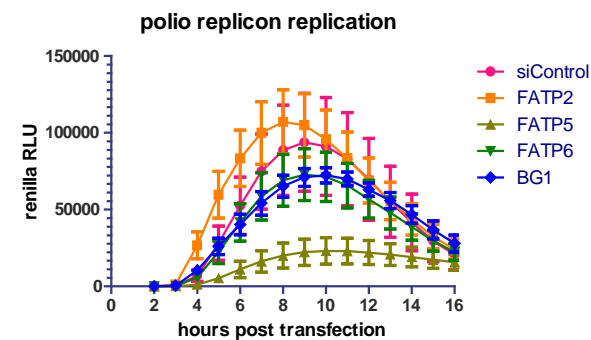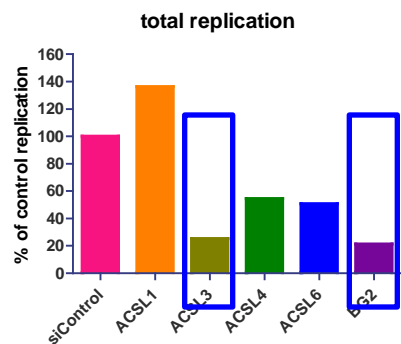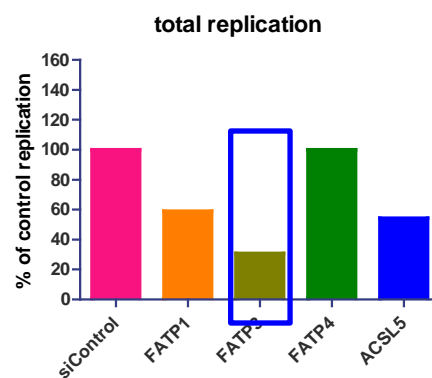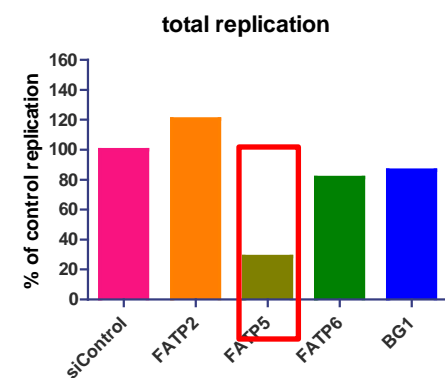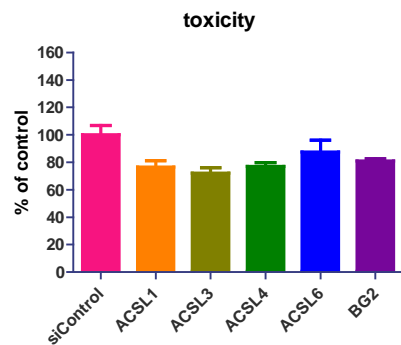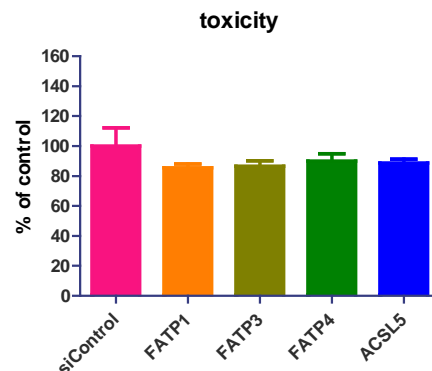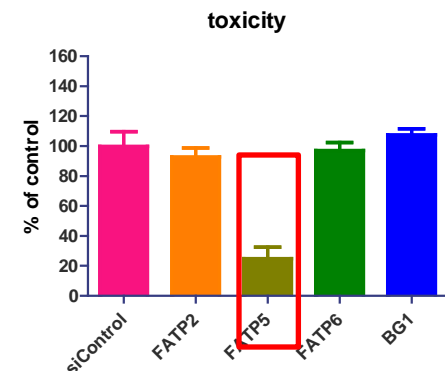

Supplement: Figure S3 — HeLa cells grown on 96 well plates were transfected with siGenome siRNA pools targeting all known human long and very long chain acyl-CoA synthetases, 16 wells for each siRNA pool. siControl scrambled siRNA (Dharmacon) served as a control. After 72 h incubation with siRNA polio replicon replication assay was performed. Total replication is calculated as area under curve using Prizm software and the data are displayed as percentage of control. Toxicity was measured after replicon replication assay. siRNAs exhibited the strongest effect on replication (Acsl3, Acs BG2 and FATP3) are outlined in blue boxes. The toxic FATP5 siRNA is outlined by the red box. (PDF) [file ppat.1003401.s003.pdf]

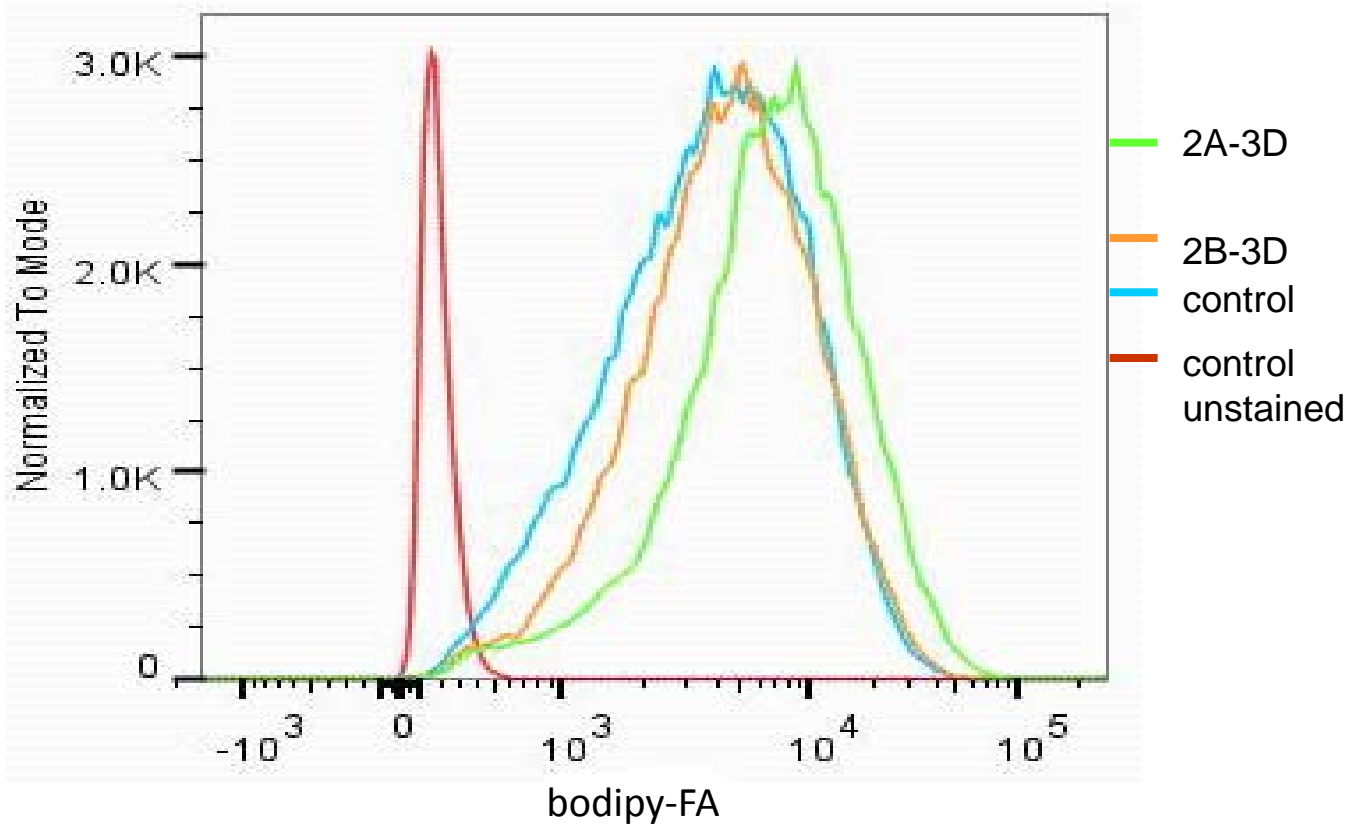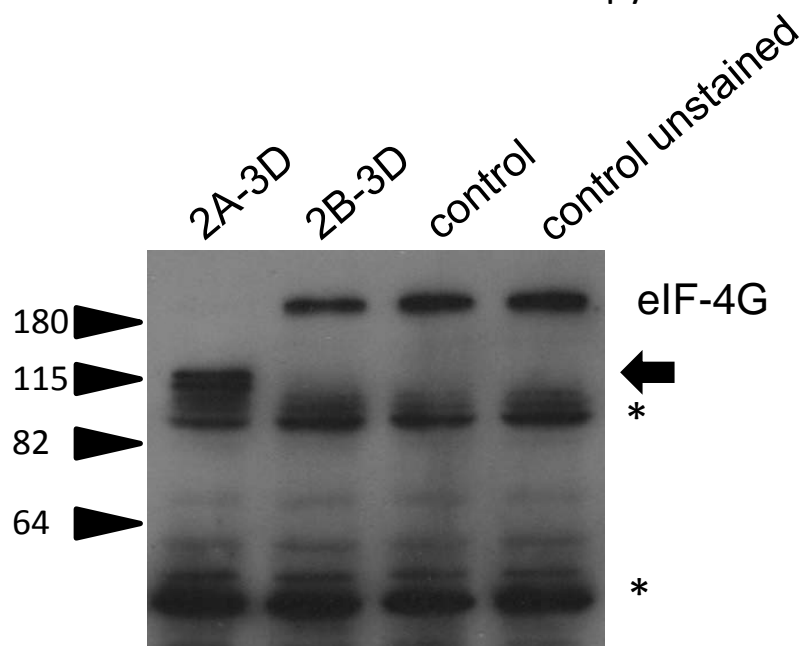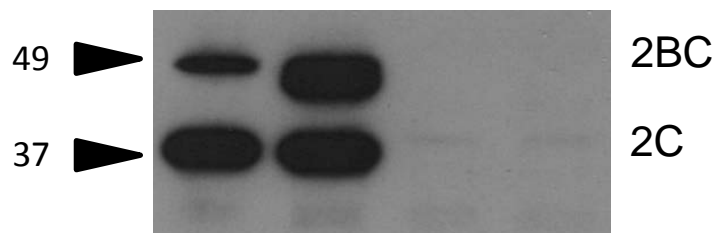

Supplement: Figure S5 — HeLa cells were transfected with plasmids coding for the indicated polyprotein fragments under control of T7 promoter (empty vector for the control sample). The next day the cells were infected with vaccinia-T7 virus and labeled with bodipy-FA for 30 min at 4 h p. i. (one control sample was incubated without bodipy-FA to measure the background cell fluorescence). After that the cells were collected, fixed in 1% PFA and processed for FACS analysis (10000 cells per sample). Aliquots of the corresponding samples were analyzed for 2A protease activity (middle panel) and expression of the viral proteins (lower panel). Processing of eIF-4G (black arrow) is detected only in the sample expressing functional 2A protease. Asterisks (*) indicate background bands showing equal amount of the loaded material. (PDF) [file ppat.1003401.s005.pdf]
