# Supplementary material for: Increased Long Chain acyl-Coa Synthetase Activity and Fatty Acid Import Is Linked to Membrane Synthesis for Development of Picornavirus Replication Organelles
Source: PLoS Pathog. 2013 Jun 6;9(6):e1003401. doi: 10.1371/journal.ppat.1003401 (PMC3675155; doi:10.1371/journal.ppat.1003401)

Inhibition of polio replication by knock-down of Acsl3 expression

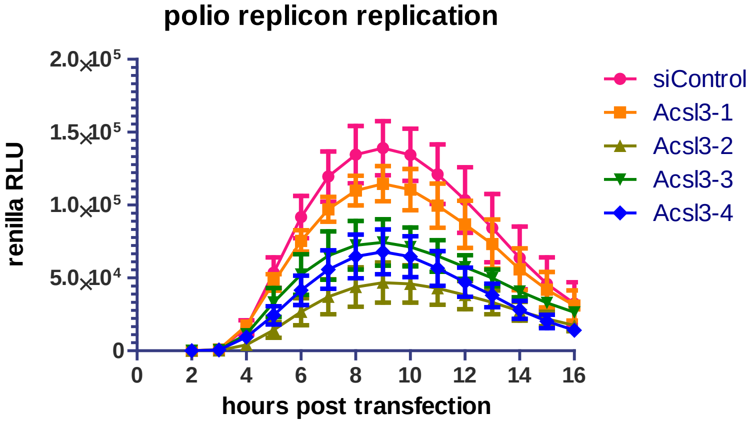

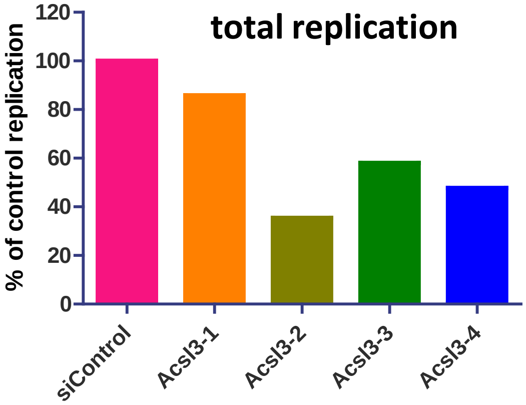

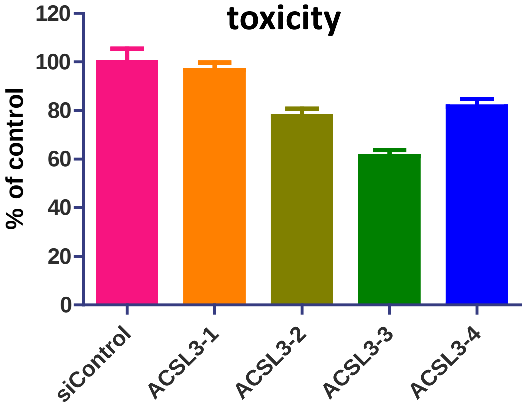

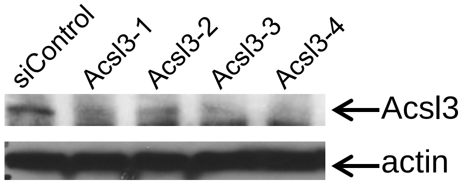

rescue of the inhibitory effect of anti-Acsl3 siRNA#2 by expression of a resistant Acsl3

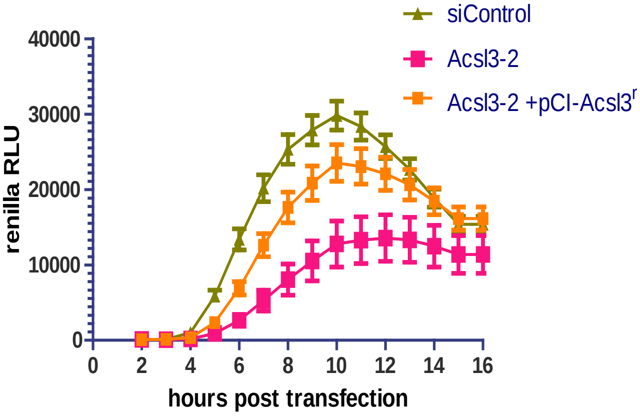

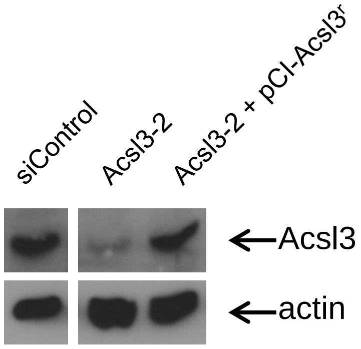

Supplement: Figure S4 — HeLa cells grown on 96 well plates were transfected with each of four individual siRNAs from the siGenome pool targeting long chain acyl-CoA synthetase 3 (Acsl3), 16 wells for each siRNA. siControl scrambled siRNA (Dharmacon) served as a control. After 72 h incubation with siRNA polio replicon replication assay was performed. Total replication is calculated as area under curve using Prizm software and the data are displayed as percentage of control. Toxicity and western blot analysis were performed after replicon replication assay. For the siRNA rescue experiment cells were transfected with the most potent anti-Acsl3 siRNA #2 (or control siRNA) and in ∼48 hours they were transfected with pCI-Acsl3r plasmid coding for the Acsl3 sequence with mutated siRNA targeting site (control samples were transfected with an empty vector). The next day after DNA transfection polio replicon assay was performed. (PDF) [file ppat.1003401.s004.pdf]
